# Infections associated with clozapine: a pharmacovigilance study using VigiBase^®^

**DOI:** 10.3389/fphar.2023.1260915

**Published:** 2023-10-02

**Authors:** Basile Chrétien, Perrine Brazo, Angélique Da Silva, Marion Sassier, Charles Dolladille, Véronique Lelong-Boulouard, Joachim Alexandre, Sophie Fedrizzi

**Affiliations:** ^1^ Department of Pharmacology, Caen University Hospital, Caen, France; ^2^ Department of Psychiatry, Esquirol Center, Caen University Hospital, Caen, France; ^3^ Normandie Université, UNICAEN, EA7466, Imagerie et Stratégies Thérapeutiques de la Schizophrénie (ISTS), Caen, France; ^4^ Normandy University, UNICAEN, Inserm U1086 Anticipe, Caen, France; ^5^ Normandy University, UNICAEN, UFR Santé, INSERM UMR 1075, COMETE-MOBILITES “Vieillissement, Pathologie, Santé”, Caen, France

**Keywords:** clozapine, infection, overdose, interaction, pharmacovigilance

## Abstract

**Introduction:** Clozapine is primarily reserved for treatment-resistant schizophrenia due to safety concerns associated with its use. Infections have been reported with clozapine, which may lead to elevated serum levels of the drug. However, the existing literature on this topic is limited. Therefore, we conducted a study using VigiBase^®^ to investigate the potential over-reporting of infections associated with clozapine, to explore the presence of dose-dependency, and to investigate the underlying mechanism.

**Methods:** Disproportionality analyses were performed using VigiBase to assess the association between clozapine and all types of infections, the association between clozapine-associated infections and neutropenia, the association between clozapine-associated infections and agranulocytosis, the dose–effect relationship between clozapine and infections, and the interaction between clozapine and the main strong CYP450 inhibitors using reports carried out until 11 April 2023.

**Results:** A statistically significant signal of infections was observed with clozapine, as indicated by an information component of 0.43 [95% CI: (0.41–0.45)]. The most commonly reported infections were respiratory and gastrointestinal in nature. Neutropenia showed weaker association with clozapine-associated reports of infections compared to other clozapine-associated reports [X2 (1, N = 204,073) = 454; *p* < 0.005], while agranulocytosis demonstrated a stronger association with clozapine-associated reports of infections [X2 (1, N = 204,073) = 56; *p* < 0.005]. No evidence of dose-dependency was observed. Among the 17 tested CYP inhibitors, significant drug–drug interactions were found with clarithromycin, metronidazole, valproic acid, lansoprazole, omeprazole, amiodarone, and esomeprazole.

**Discussion:** Our study revealed a significant safety signal between clozapine use and infections, predominantly respiratory and gastrointestinal infections. The co-administration of clozapine with valproic acid or proton pump inhibitors may potentially contribute to an increased risk of infection. Further vigilance is warranted in clinical practice, and consideration of therapeutic drug monitoring of clozapine in cases involving concomitant use of these drugs or in the presence of infections may be beneficial.

## Introduction

Clozapine is an atypical antipsychotic drug mostly used in patients with treatment-resistant schizophrenia. Over the years, its use has increased in many countries, with the prevalence of clozapine consumption in 2014 ranging from 0.9 to 173.2 per 100,000 persons, depending on the country (Bachmann et al., 2017; Remington et al., 2017). Safety issues associated with clozapine are common and include agranulocytosis (occurring in approximately 1.0% of patients) and neutropenia (occurring in approximately 3.0% of patients) (Rajagopal, 2005). Due to these safety concerns, clozapine is usually reserved for treatment-resistant schizophrenia despite its demonstrated efficacy in managing positive, negative, and overall symptoms and relapse rates in schizophrenia, compared to first-generation antipsychotics and pooled first-/second-generation antipsychotics (Wagner et al., 2021).

Infections have also been reported with clozapine, potentially leading to the elevation of serum levels of the drug. A systematic review identified 40 cases of infections with demonstrated elevated clozapine levels (Clark et al., 2018). In a Chinese cohort of patients undergoing therapeutic drug monitoring during infection and non-infection periods, the median levels of clozapine were significantly higher in the infection period compared to those in the non-infection period (*n* = 42; *p* < 0.001) (Zhang et al., 2021). Elevation of clozapine levels with infection may be due to downregulation of metabolizing enzymes such as cytochrome P450 (Clark et al., 2018). Additionally, a retrospective study from the UK found an increased risk of COVID-19 infection in clozapine-treated patients compared to those on other antipsychotic drugs (adjusted hazard ratio = 1.76; 95% CI 1.14–2.72) (Govind et al., 2021).

Clozapine might be more strongly associated with pneumonia than with other infections. A study on a Taiwanese registry with 33,024 inpatients with schizophrenia found that the current use of clozapine was associated with a dose-dependent increase in the risk of pneumonia (adjusted risk ratio = 3.18; 95% CI: 2.62–3.86) (Kuo et al., 2013). A study using VigiBase^®^, the WHO global safety report database, supports the prominent role of pneumonia in mortality associated with clozapine adverse drug reactions (De Leon et al., 2020).

Patients with schizophrenia often receive polypharmacotherapy, exposing them to potential drug–drug interactions (DDIs). A cross-sectional observational study in a psychiatric hospital found a high prevalence (88.7%) of potential DDI in this population (Bačar Bole et al., 2023). Clozapine is metabolized by various CYP450 enzymes, including CYP1A2, CYP3A4, CYP2D6, CYP2C9, and CYP2C19 (Chetty and Murray, 2007; Pardiñas et al., 2019). Therefore, the association of clozapine with CYP450 inhibitors could lead to clozapine overdose and increase the risk of infection.

Sex-related differences in clozapine tolerability have also been described in 147 treatment-resistant patients treated with clozapine (Martini et al., 2021). Age onset incidence of schizophrenia also differs by sex (Jauhar et al., 2022). As sex-specific differences in the susceptibility to infections and immune response to infection have been reported (McClelland and Smith, 2011), it is also possible that sex differences in clozapine-associated infections exist.

However, until now, studies about clozapine-associated infections remain scarce and are poorly described. Except for clozapine-induced pneumonia, which has been widely described (De Leon et al., 2020), it is unclear what kind of other infection clozapine exposure might lead to. The mechanism behind these infections also needs to be understood as it could be hypothesized that clozapine-induced infections are caused by clozapine-associated neutropenia. Dose-dependency, patient-associated risk factors, and potential DDIs leading to infections also need to be explored to improve preventive measures. Thus, for a better understanding of clozapine-associated infections, a study was conducted using VigiBase^®^. The objectives were to assess if clozapine was associated with an over-reporting of infections, to characterize these infections, to evaluate dose-dependency and sex differences, and to investigate potential DDIs associated with infections. The study also explored if clozapine-associated infections were more associated with neutropenia.

## Materials and methods

### Study design

We conducted a retrospective pharmacovigilance cohort study using VigiBase^®^, the WHO global individual case safety report (ICSR) database. Access to the data was granted by the WHO Uppsala Monitoring Centre. The database contains suspected drugs, suspected adverse drug reactions (ADRs), patient demographics, and other variables, with over 32 million ICSRs received from 130 countries, since 1967 and previously described elsewhere (Chrétien et al., 2021). The study protocol was registered on ClinicalTrials.gov with the identifier NCT05919550.

### Setting and participants

We utilized all the reports from the de-duplicated VigiBase^®^ dataset from the 11th of April 2023 version. The suspected duplicates were identified using vigiMatch, an algorithm developed by the Uppsala Monitoring Centre, and excluded. The scope of the dataset was limited to drugs; vaccines were excluded due to their distinct usage profile, potential for higher infection reporting, and reporting bias associated with the COVID-19 pandemic.

### Variables

Infections were identified using the Medical Dictionary for Regulatory Activities (MedDRA v25.1) System Organ Class infections and infestations (Supplementary Table S1). The analysis included preferred terms of infection, as shown in Supplementary Table S1. Some terms were overly general, making it challenging to comprehensively understand the nature of the ongoing infection (e.g., the term “Infection”). Neutropenia and agranulocytosis were defined using their associated preferred terms. Clozapine was identified using the Anatomical and Therapeutic Chemical classification and/or its international non-proprietary name. The daily dose of clozapine was extracted from clozapine reports and divided into quartiles to perform the dose-dependency analysis (reports with the available daily dose were attributed to their corresponding quartile).

### Outcomes

The primary outcome was the association between clozapine and all types of infections. Secondary outcomes included the following:

- The association between clozapine and all types of infections over time (cumulative).
- The association between clozapine and all types of infections:
   o In women population
   o In men population
   o In people aged 44 or lower
   o In people aged 45 or higher
- The association of clozapine with more detailed terms of infection (all MedDRA preferred terms included in the infection and infestation System Organ Class groups), for which at least five cases were reported with clozapine.
- The association between clozapine-associated infection and neutropenia, and the association between clozapine-associated infection and agranulocytosis.
- The dose–effect relationship between clozapine and infections.
- The interaction between clozapine and various strong CYP450 inhibitors [amiodarone, atazanavir, cannabidiol, ciclosporin, clarithromycin, clobazam, esomeprazole, felbamate, fluconazole, fluvoxamine, itraconazole, lansoprazole, metronidazole, omeprazole, ritonavir, voriconazole, and valproic acid (VPA)] and the reporting of infection. CYP450 inhibitors were defined using WHODrug Insight (Uppsala Center, 2023) and the interaction table from Geneva University Hospitals (Geneva University Hospital, 2020) (Supplementary Table S3).

### Statistical analyses

To assess the association of clozapine with the reporting of infections, a disproportionality analysis was used to evaluate the associations between drugs and reactions using VigiBase^®^ (Faillie, 2019). This type of study was previously described (Chrétien et al., 2021). In the present study, the information component (IC) and its 95% credibility interval (CI) were used to evaluate disproportionality. The IC is a Bayesian measure of the disproportionality between the observed and the expected reporting of a drug–ADR pair, developed by members of the WHO Uppsala Monitoring Centre (Bate et al., 1998). We chose to compute the analysis only for drugs that reported at least five cases of infections as IC was found to be more reliable when at least three to five cases of an ADR were reported for a drug (Evans et al., 2001). A lower end of the 95% CI of the IC > 0 was deemed significant.

- A disproportionality analysis was used to evaluate the association between clozapine and all MedDRA preferred terms included in the infection and infestation System Organ Class groups using the same method as described previously.
- A disproportionality analysis was used to evaluate the association between each quartile of the dose of clozapine and the reporting of infections using the same method as described previously.
- A disproportionality analysis was used to evaluate the association between clozapine and various CYP450 inhibitors (amiodarone, atazanavir, cannabidiol, ciclosporin, clarithromycin, clobazam, esomeprazole, felbamate, fluconazole, fluvoxamine, itraconazole, lansoprazole, metronidazole, omeprazole, ritonavir, voriconazole, and VPA) and the reporting of infection. IC was also used for this analysis.
- A chi-squared test was used to assess the association between clozapine-associated infection and neutropenias by comparing the reporting of neutropenia in clozapine-associated infection reports to the reporting of neutropenia in other clozapine-associated reports. The same test was used with agranulocytosis.

### Descriptive study using VigiBase^®^

We described the clinical features of clozapine-related infections, reporting the reports’ completeness score, demographic parameters (age and sex), dose, seriousness, and percentage of death. The percentage of seriousness and death with other drugs associated with infections was also evaluated for comparison.

## Results

### Statistical analysis to assess the association of clozapine with the reporting of infections

**FIGURE 1:**
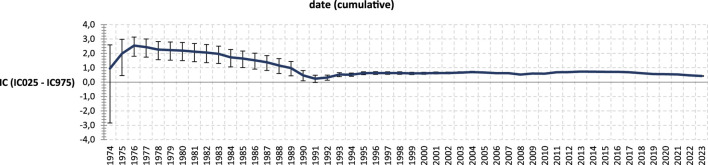
Disproportionality analysis of clozapine-associated infection over time (cumulative*). *Cumulative: previous data are used at the current date. For example, for the year 1977, data from 1974–1977 are used to compute the disproportionality analysis. Thus, at each point of the figure, cases and non-cases reported until this point are used to compute the information component.

Out of the 204,073 reports of clozapine-associated suspected ADRs, 19,404 were related to infections. A statistically significant signal of infections was found with clozapine, with an IC of 0.43 [95% CI: (0.41–0.45)] (Table 1). This signal was of similar magnitude among genders and tested age classes (Table 1). It was also consistent over time (Figure 1).

**TABLE 1:** Disproportionality analysis in VigiBase: reports of association of clozapine with infections.

	N	IC	95% CI
Clozapine	19,404	0.43	[0.41; 0.45]
Clozapine (men)^a^	11,585	0.54	[0.51; 0.56]
Clozapine (women)^a^	7,534	0.44	[0.40; 0.47]
Clozapine (<45 yo)^a^	6,102	0.25	[0.21; 0.28]
Clozapine (≥45 yo)^a^	9,577	0.71	[0.68; 0.73]

N, number of reports; IC, information component; 95% CI, 95% credibility interval; yo, year old.

^a^ Age was unknown in 19.8%, and sex was unknown un 2.6%.

### Descriptive study using VigiBase^®^

Table 2 presents the features of clozapine-associated reports of infections. Among these reports, 94.3% were considered serious and 9.8% of cases resulted in death. This is compared with reports of infection associated with other drugs, where 62.6% were considered serious and 6.8% resulted in death.

**TABLE 2:** Description of clozapine-associated reports of infection.

Age	N available	15,549
	Median (IQR) in years	50 (38–62)
Sex	N available	18,906
	Sex ratio (male/female individuals)	1.57
Dose (in mg)	N available	2,319
	Median (IQR)	250 (100–400)
Completeness score (median with IQR)	0.42 (0.28–0.62)	
Seriousness	N available	15,421
	%	94.3%
Death (%)	9.8%	

### Statistical analysis to assess the association of clozapine with all MedDRA preferred terms included in the infection and infestation System Organ Class groups

A total of 60 terms of infection were significantly associated with clozapine. The four most reported terms were pneumonia (5,751 reports), lower respiratory tract infection (2,268 reports), urinary tract infection (1,628 reports), and infections (1,564 reports). The 10 most significant signals were pneumonia aspiration [N = 850; IC: 3.28 (3.17–3.37)], viral myocarditis [N = 25; IC: 3.64 (2.97–4.11)], empyema [N = 75; IC = 3.22 (2.83–3.49)], lower respiratory tract infection [N = 2,268; IC: 2.79 (2.72–2.84)], infective exacerbation of chronic obstructive airways disease [N = 30; IC: 3.24 (2.63–3.67)], appendicitis perforated [N = 93; IC: 2.81 (2.46–3.05)], appendicitis [N = 261; IC: 2.61 (2.41–2.76)], abdominal sepsis [N = 24; IC: 3.01 (2.32–3.49)], lung abscess [N = 58; IC: 2.69 (2.25–3.00)], and complicated appendicitis [N = 9; IC: 3.18 (2.04–3.94)] (Table 3; Supplementary Table S5).

**TABLE 3:** Terms of infection associated with a significant over-reporting with clozapine (more than 15 reports per term).

Term	N	IC	IC025^a^	IC975
Pneumonia aspiration	850	3.28	3.17	3.37
Viral myocarditis	25	3.64	2.97	4.11
Empyema	75	3.22	2.83	3.49
Lower respiratory tract infection	2,268	2.79	2.72	2.84
Infective exacerbation of chronic obstructive airways disease	30	3.24	2.63	3.67
Appendicitis perforated	93	2.81	2.46	3.05
Appendicitis	261	2.61	2.41	2.76
Abdominal sepsis	24	3.01	2.32	3.49
Lung abscess	58	2.69	2.25	3.00
Parotitis	41	2.36	1.84	2.73
Coronavirus infection	178	2.07	1.82	2.25
Chronic hepatitis C	15	2.55	1.68	3.15
Pneumonia	5,751	1.67	1.62	1.70
Hepatitis C	302	1.73	1.54	1.87
Sialadenitis	43	2.05	1.54	2.41
Urosepsis	149	1.80	1.53	2.00
H1N1 influenza	30	2.02	1.41	2.45
Sepsis	1,521	1.43	1.35	1.49
Infectious pleural effusion	23	2.02	1.32	2.51
Viral infection	489	1.29	1.14	1.4
Suspected COVID-19	62	1.53	1.11	1.83
Gastroenteritis	225	1.25	1.03	1.41
Pulmonary sepsis	16	1.74	0.90	2.33
Infection	1,564	0.96	0.88	1.02
Respiratory tract infection	257	1.07	0.87	1.22
Stoma site infection	25	1.46	0.79	1.93
Neutropenic sepsis	65	1.19	0.78	1.49
Urinary tract infection	1,628	0.76	0.68	0.82
Endocarditis	51	1.14	0.67	1.47
Pyelonephritis	83	0.87	0.51	1.13
Hepatitis viral	15	1.35	0.48	1.95
Brain abscess	19	1.24	0.47	1.78
COVID-19	1,155	0.54	0.45	0.61
Pharyngitis	256	0.64	0.43	0.79
Abscess oral	22	1.11	0.39	1.61
Atypical pneumonia	34	0.91	0.34	1.32
Infectious mononucleosis	26	1.00	0.34	1.46
Septic shock	187	0.43	0.19	0.6
Encephalitis	42	0.67	0.16	1.03
Abscess	142	0.37	0.09	0.57

N, number of reports; IC, information component; IC025, lower end of the credibility interval; IC975, upper end of the credibility interval.

^a^ The lower end of the credibility interval (IC025) is traditionally used as the threshold for generating a safety signal in pharmacovigilance databases and was used here to order the table.

### Statistical analysis to assess the association of clozapine-associated reports of infections with neutropenia or agranulocytosis

Neutropenia was reported in 6.13% of the reports of clozapine-associated infections. It was less associated with clozapine-associated reports of infections compared to other clozapine-associated reports: X2 (1, N = 204,073) = 454; *p* < 0.0005). However, agranulocytosis was more associated with clozapine-associated reports of infections compared to other clozapine-associated reports: X2 (1, N = 204,073) = 56; *p* < 0.0005. However, agranulocytosis was reported in only 3.33% of the reports of clozapine-associated infection.

### Statistical analysis to assess the dose–effect relationship between the dose of clozapine and infections

The dose was rarely reported as only 2,319 (1.14%) reports included this information. When using the dose to perform the disproportionality analysis, no signal of dose-dependency could be found (Supplementary Table S4). The quartiles of doses found in our study were as follows: 100 mg/day, 250 mg/day, and 400 mg/day.

### Statistical analysis to assess the DDI between clozapine and various CYP450 inhibitors on the reporting of infections

Among the 17 tested CYP inhibitors, a significant drug–drug interaction was found with clarithromycin, metronidazole, VPA, lansoprazole, omeprazole, amiodarone, and esomeprazole (Table 4).

**TABLE 4:** Disproportionality analysis for the drug–drug interaction between clozapine and a list of CYP450 inhibitors on the reporting of infections.

CYP inhibitor	N	IC	IC025	IC975	CYP1A2		CYP3A4		CYP2D6		CYP2C9		CYP2C19	
					WHO	HUG	WHO	HUG	WHO	HUG	WHO	HUG	WHO	HUG
Clarithromycin^a^	65	1.99	1.58	2.29	—	—	—	ST	—	—	—	—	—	—
Metronidazole^a^	53	1.68	1.22	2.01	—	—	—	—	—	—	WE	ST	—	—
Valproic acid^a^	1944	0.96	0.89	1.02	—	—	—	—	—	—	UN	ST	—	—
Lansoprazole^a^	453	0.95	0.8	1.07	—	—	—	—	—	—	—	—	UN	ST
Omeprazole^a^	598	0.49	0.36	0.59	—	—	—	—	—	—	—	—	MO	ST
Amiodarone^a^	15	1.18	0.31	1.78	UN	MO	WE	MO	WE	ST	MO	ST	—	—
Esomeprazole^a^	136	0.49	0.2	0.69	—	—	—	—	—	—	—	—	MO	ST
Clobazam	18	0.78	−0.01	1.33	—	—	—	MO	WE	—	—	—	—	MO
Fluvoxamine	45	0.43	−0.07	0.78	ST	ST	MO	MO	UN	—	WE	MO	ST	ST
Fluconazole	30	0.28	−0.33	0.71	—	—	MO	ST	—	—	MO	ST	ST	ST
Ciclosporin	8	−0.25	−1.46	0.55	—	—	MO	ST	—	—	—	—	—	—
Voriconazole	3	NA	NA	NA	—	—	ST	ST	—	—	WE	ST	MO	ST
Itraconazole	3	NA	NA	NA	—	—	ST	ST	—	—	—	—	—	—
Ritonavir	2	NA	NA	NA	UN	—	ST	ST	WE	ST	UN	—	—	—
Cannabidiol	1	NA	NA	NA	—	MO	—	ST	—	—	—	—	—	—
Felbamate	0	NA	NA	NA	—	—	—	—	—	—	—	—	WE	ST
Atazanavir	0	NA	NA	NA	UN	MO	—	ST	—	—	UN	MO	—	—

CYP, cytochrome; FDA, Food and Drug Administration; HUG, Hôpital Universitaire de Genève (Geneva University Hospitals); MO, moderate inhibitor; N, number of reports; NA, not applicable (not enough reports to compute the analysis); IC, information component; IC025, lower end of the credibility interval, IC975, upper end of the credibility interval; ST, strong inhibitor; UN, unclassified inhibitor; WE, weak inhibitor; WHO, World Health Organization classification.

HUG, classification contains two levels: MO and ST.

WHO, classification contains four levels: UN, WE, MO, and ST.

^a^ Statistically significant signal of the drug–drug interaction.

## Discussion

In our study, conducted under real-life conditions using the largest pharmacovigilance database worldwide, we identified a significant association between clozapine and reported infections. This signal was consistent over time (Figure 1). Various types of infections were linked to clozapine use in our study, with 94.3% of them considered serious and 9.8% resulting in death at the time of reporting. Although serious adverse effects tend to be more reported than non-serious effects, our findings imply that people treated with clozapine should be carefully followed in the case of symptoms of infection.

The most frequently reported infections were respiratory and gastrointestinal in nature. Patients and families should be educated about the risk of infections, particularly respiratory and gastrointestinal infections.

Clozapine may be particularly associated with pneumonia compared to other infections. A Taiwanese registry study involving a nationwide cohort of 33,024 inpatients with schizophrenia found that the current use of clozapine (adjusted RR = 3.18; 95% CI: 2.62–3.86, *p* < 0.001) was linked to a dose-dependent increase in the risk of pneumonia. On the other hand, quetiapine, olanzapine, zotepine, and risperidone showed a lesser extent of association with an increased risk of pneumonia. Unlike clozapine, no clear dose-dependent relationship was observed for these antipsychotics. The risk of pneumonia with clozapine was found to be strongest within the first 30 days of treatment (Kuo et al., 2013).

A retrospective study based on clinical records from the UK also reported an increased risk of COVID-19 infection in clozapine-treated patients compared to those on other antipsychotic medications (adjusted HR = 1.76; 95% CI 1.14–2.72) (Govind et al., 2021). However, a register-based cohort study from the Stockholm region did not find an association between clozapine treatment and severe COVID-19 infection. The adjusted HR for the exposed group compared to the unexposed group was 0.96 (95% CI: 0.54 and 1.70) for inpatient care, 1.69 (0.48 and 5.93) for care in an intensive care unit (ICU), and 0.86 (0.26 and 2.80) for death (Ohlis et al., 2022). This study might have lacked statistical power due to the low number of clozapine-treated patients suffering from COVID-19 during the study period. In another English retrospective 1-year cohort study, the incidence of infection was compared between 64 patients starting clozapine and 120 patients starting paliperidone palmitate long-acting injection (PPLAI). The incidence of infection was greater in clozapine starters than in PPLAI starters (28% vs. 6%; *p* = 0.001; adjusted odds ratio 5.82 (95% CI = 2.15–15.76). Similar to our findings, infectious episodes in clozapine patients were not statistically related to changes in neutrophil counts. According to the authors’ classification, the most commonly reported infection in the clozapine group was respiratory infection; however, the majority of infections were non-respiratory-related (Mace et al., 2022).

Regarding the risk of gastrointestinal infection, particularly appendicitis, a retrospective study including 465 patients, of whom 65 were on clozapine, showed that the clozapine exposure group exhibited a higher incidence of appendicitis during the observation period than the non-exposure group (863 cases vs. 124 cases per 100,000 person-years) (Kawakita et al., 2022). Additionally, a case series of six patients with perforated appendicitis during clozapine treatment reported a 20-fold increase in appendicitis incidence compared to the general population in male subjects of the same age group (Steinert and Jans, 2021). The authors also suspected a dose-dependency as they observed high clozapine serum levels in three of those patients during the course of appendicitis.

Possible indirect mechanisms of clozapine predisposition to infection, particularly aspiration pneumonia, include sialorrhea and impairment of swallowing function with esophageal dilatation and hypomotility (Abdelmawla and Ahmed, 2009). A systematic review during the first month of clozapine treatment indicated that up to 50% of patients develop fever and flu-like symptoms, seemingly driven by increased cytokines (Røge et al., 2012). A recent study comparing the levels of secondary antibodies of clozapine users with those of the users of antipsychotics other than clozapine (control group) noted lower secondary antibodies among clozapine users. Total serum immunoglobulins [immunoglobulin (Ig)G, IgA, and IgM] and specific immunoglobulin antibodies to *Haemophilus influenzae* and pneumococcus were decreased (Ponsford et al., 2019). Lower immunoglobulin levels might contribute to the onset of infections.

In our study, neutropenia was less associated with clozapine-associated reports of infections compared to other clozapine-associated reports (*p*-value < 0.005), indicating that most of these infections were probably not linked to clozapine-associated neutropenia. However, agranulocytosis was more associated with clozapine-associated reports of infections compared to other clozapine-associated reports (*p*-value < 0.005), which is expected as infection is very common in agranulocytosis patients. Indeed, a retrospective Finn study on 163 patients with clozapine-induced agranulocytosis found that 78.6% of the patients presented with an infection (Lahdelma and Appelberg, 2012). The median age of the patients in this study was 49 years, which is very close to the median age in our study (50 years), but the sex ratio was lower in their study (1.09 vs. 1.57 in our study). However, since agranulocytosis was reported in only 3.33% of the reports of clozapine-associated infections, it is not the main cause of the signal of infections observed in our study.

Although men and women have a similar prevalence of schizophrenia (Li et al., 2016), research indicates that men often experience the onset of severe schizophrenia symptoms at an earlier age compared to women. Specifically, men tend to encounter the peak period of initial pronounced psychotic symptoms between 20 and 24 years of age, whereas women tend to experience these symptoms 5 or more years later (Kahn et al., 2015). This might be the reason why there are important gender differences in clozapine prescription. Indeed, several studies, including ours, have reported that men represented between 63.1% and 78.6% of patients treated with clozapine (Taylor, 2004; Silveira et al., 2015). Consequently, sex is probably not a risk factor for clozapine-induced infections.

The median dose in our study was 250 mg (IQR = 100–400). This aligns with the typical dose found in the clozapine-treated population as Caucasians are usually prescribed 300–600 mg/day to reach the therapeutic range, while in Asian countries, average clozapine doses are lower than 300 mg/day (de Leon et al., 2020). However, it is essential to consider that dose reporting was unavailable in more than 98% of the reports in the database. The results of the tests performed in this study to explore a dose-dependency effect do not allow us to draw a conclusion.

The results of the disproportionality analysis for drug–drug interactions (DDI) between clozapine and CYP450 inhibitors on the reporting of infections are more challenging to interpret. Except for ciclosporin (an inhibitor of CYP3A4), a trend toward a DDI was found with all CYP inhibitors, for which a disproportionality analysis could be computed. However, it is unclear which specific CYP450 enzymes may be involved. The associations observed with certain CYP inhibitors, such as clarithromycin and metronidazole, may be influenced by an indication bias since these drugs are commonly prescribed in the context of infections. VPA, one of the most co-reported drugs in the DDI analysis, has been associated with an increase in oxidative stress. Given the potential for clozapine to induce oxidative stress as well, the combination of VPA and clozapine may potentially favor the onset of certain types of infections (Gai et al., 2020; Salimi et al., 2022).

Some authors suspect that the association of clozapine with proton pump inhibitors (PPI) might increase the formation of reactive metabolites and contribute to the increase in hematological adverse drug reactions (ADRs) (Wiciński et al., 2017). However, the oxidative stress hypothesis is inconclusive with PPIs as these drugs usually alleviate oxidative stress (Gao et al., 2019; Gandhi et al., 2021). PPIs have been associated with respiratory infections in different retrospective studies, suggesting a potential pharmacodynamic interaction (Ho et al., 2017; Wang et al., 2019). In a retrospective chart review, aiming to explore the potential effect of polypharmacy on the hematologic profiles of clozapine patients, 24 out of 26 (96%) of the subset of patients who were prescribed a PPI or ranitidine concomitantly with clozapine experienced hematological ADRs (Shuman et al., 2014). A case of infection during PPI treatment with elevated plasma clozapine levels was reported in a 51-year-old woman and could potentially be linked to the switch from omeprazole to esomeprazole (Wagner et al., 2011). The authors speculated that this might be due to the removal of induction of clozapine metabolism by omeprazole. However, the delay was not in favor of this hypothesis. Omeprazole and lansoprazole, in addition to being CYP2C19 inhibitors, are also CYP1A2 inducers. In a case series involving two patients, the prescription of omeprazole was associated with a reduction in clozapine plasma concentrations of 41.9% and 44.7% (Frick et al., 2003). Another retrospective study in 13 psychiatric patients found that the switch from omeprazole to pantoprazole led to an increase in clozapine levels in non-smoking patients and to a decrease in clozapine levels in smoking patients. This was probably caused by the discontinuation of enzyme induction in the cytochrome P450 enzyme 1A2 by omeprazole in non-smokers, whereas CYP1A2 remained induced in smokers (Mookhoek and Loonen, 2004). As the prevalence of cigarette smoking is high in schizophrenia patients (around 70%–80%) and probably even higher in treatment-resistant schizophrenia patients (Ding and Hu, 2021), we believe that the DDI observed in our study between PPIs and clozapine is probably more linked to the CYP2C19 inhibition by PPIs.

Regardless of the cause of infection, several reports showed that infection leads to an increase in the toxicity levels of clozapine and its metabolites in the serum (Darling and Huthwaite, 2011; Espnes et al., 2012; Zhang et al., 2021; Chengappa et al., 2022). This is likely to be mediated by cytokine suppression of cytochrome P450 1A2 (CYP1A2), the main hepatic microsomal system involved in clozapine metabolism, which is also involved in the metabolism of several antibiotics commonly used to treat infections. This further enhances the potential for clozapine toxicity (Røge et al., 2012). However, despite sex being a factor of cytochrome P450 expression (Zanger and Schwab, 2013), we did not find any sex-related significant differences in clozapine-associated infections.

### Limitations

Our study was subject to various inherent limitations stemming from the utilization of a pharmacovigilance database. Of paramount importance among these limitations is the issue of under-reporting. Nevertheless, it is reassuring that despite this limitation, the results and significance of the disproportionality analysis remained unaffected (Montastruc et al., 2011).

However, it is crucial to recognize that the likelihood of a suspected ADR being drug-related may not be uniform across all cases. Although disproportionality analysis of spontaneous reports is a valuable tool for detecting safety signals, it possesses certain intrinsic limitations as well. One such limitation is the potential presence of low-quality data due to missing information. Additionally, the causal relationship between the reported drug and the ADR remains unproven (Egberts et al., 2002; Garbe and Suissa, 2014).

Moreover, it is worth noting that the reporting pattern of ADRs may vary between new and old drugs, with more rigorous monitoring typically occurring during the period of drug marketing and shortly thereafter.

It is vital to emphasize that, due to under-reporting of ADRs, pharmacovigilance data cannot be utilized to determine the incidence rates of ADRs (Sharrar and Dieck, 2013). Furthermore, our study lacked information on the smoking history of participants despite its significant impact on the pharmacokinetics of clozapine and the risk of respiratory infection.

## Conclusion

This study has revealed a significant safety signal concerning the association between clozapine and reported infections. Respiratory infections, as well as gastrointestinal infections, including appendicitis, were the most commonly reported infections. The co-administration of clozapine with valproic acid (VPA) or proton pump inhibitors (PPIs) may potentially increase the risk of infection. However, as this study was based on pharmacovigilance data, a definitive causal relationship between clozapine exposure and the occurrence of infections cannot be established with certainty. Nevertheless, in clinical practice, psychiatrists should remain vigilant for signs of infections when prescribing clozapine and ensure that mandatory vaccines, including pneumococcal vaccination, have been administered. Conducting a study to evaluate the relevance of therapeutic drug monitoring of clozapine when the patient’s treatment regimen is altered (especially with VPA or PPIs) or when an infection occurs would be of considerable interest.

## Data Availability

The datasets presented in this article are not readily available because data are owned by the WHO Uppsala Monitoring Center. Requests to access the datasets should be directed to https://who-umc.org/vigibase/.
