# Supplementary material for: Infections associated with clozapine: a pharmacovigilance study using VigiBase®
Source: Front Pharmacol. 2023 Oct 2;14:1260915. doi: 10.3389/fphar.2023.1260915 (PMC10577313; doi:10.3389/fphar.2023.1260915)
Supplement: Supplementary file 1 [file DataSheet1.docx]

Supplementary Material

Infections associated with clozapine: a pharmacovigilance study in VigiBase®

**Basile Chrétien, Marion Sassier, Charles Dolladille, Véronique Lelong-Boulouard, Joachim Alexandre, Sophie Fedrizzi**

*** Correspondence:** Basile Chrétien: [chretien-b@chu-caen.fr](mailto:chretien-b@chu-caen.fr)

Supplementary Table I: Terms of infections used in the secondary outcome: association of clozapine with precise terms of infection. Those terms were selected because at least 5 cases of clozapine associated with this term were reported in VigiBase®.

| MedDRA Preferred Term | Number of reports |
| --- | --- |
| Pneumonia | 5 751 |
| Lower respiratory tract infection | 2 268 |
| Urinary tract infection | 1 628 |
| Infection | 1 564 |
| Sepsis | 1 521 |
| COVID-19 | 1 155 |
| Pneumonia aspiration | 850 |
| Viral infection | 489 |
| Nasopharyngitis | 404 |
| Influenza | 364 |
| Cellulitis | 329 |
| Hepatitis C | 302 |
| Bronchitis | 278 |
| Appendicitis | 261 |
| Respiratory tract infection | 257 |
| Pharyngitis | 256 |
| Upper respiratory tract infection | 228 |
| Gastroenteritis | 225 |
| Septic shock | 187 |
| Coronavirus infection | 178 |
| Urosepsis | 149 |
| Abscess | 142 |
| Peritonitis | 138 |
| Appendicitis perforated | 93 |
| Staphylococcal infection | 93 |
| Bacterial infection | 91 |
| Rhinitis | 88 |
| Pyelonephritis | 83 |
| Herpes zoster | 80 |
| Tooth abscess | 80 |
| Tuberculosis | 79 |
| Empyema | 75 |
| Sinusitis | 70 |
| Localised infection | 66 |
| Cystitis | 65 |
| Neutropenic sepsis | 65 |
| Kidney infection | 64 |
| Clostridium difficile infection | 63 |
| Suspected COVID-19 | 62 |
| Lung abscess | 58 |
| Osteomyelitis | 58 |
| Ear infection | 55 |
| Endocarditis | 51 |
| Gastrointestinal infection | 50 |
| Tonsillitis | 48 |
| Gastroenteritis viral | 46 |
| COVID-19 pneumonia | 46 |
| Wound infection | 45 |
| Tooth infection | 45 |
| Sialoadenitis | 43 |
| Encephalitis | 42 |
| Parotitis | 41 |
| Skin infection | 41 |
| Furuncle | 37 |
| Subcutaneous abscess | 36 |
| Diverticulitis | 35 |
| Atypical pneumonia | 34 |
| Clostridium difficile colitis | 34 |
| Conjunctivitis | 31 |
| Fungal infection | 31 |
| Meningitis | 31 |
| Pneumonia bacterial | 31 |
| Candida infection | 31 |
| Bacteraemia | 30 |
| Infective exacerbation of chronic obstructive airways disease | 30 |
| H1N1 influenza | 30 |
| Hepatitis B | 29 |
| Infectious mononucleosis | 26 |
| Viral myocarditis | 25 |
| Abscess limb | 25 |
| Stoma site infection | 25 |
| Eye infection | 24 |
| Varicella | 24 |
| Abdominal sepsis | 24 |
| Pneumonia viral | 23 |
| Infectious pleural effusion | 23 |
| Abscess oral | 22 |
| Rash pustular | 22 |
| Viral upper respiratory tract infection | 21 |
| Anal abscess | 21 |
| Escherichia infection | 21 |
| Clostridial infection | 20 |
| Device related infection | 20 |
| Brain abscess | 19 |
| Gangrene | 19 |
| Post procedural infection | 19 |
| Otitis media | 18 |
| HIV infection | 16 |
| Oral candidiasis | 16 |
| Pulmonary sepsis | 16 |
| Abdominal infection | 16 |
| Abdominal abscess | 16 |
| Chronic hepatitis C | 15 |
| Gingivitis | 15 |
| Hepatitis viral | 15 |
| Postoperative wound infection | 15 |
| Respiratory tract infection viral | 15 |
| Pseudomembranous colitis | 14 |
| Purulent discharge | 14 |
| Febrile infection | 14 |
| Staphylococcal sepsis | 14 |
| Streptococcal infection | 14 |
| Cholecystitis infective | 14 |
| Breast abscess | 13 |
| Herpes simplex | 13 |
| Orchitis | 13 |
| Pyuria | 13 |
| Groin abscess | 13 |
| Arthritis bacterial | 13 |
| Pseudomonas infection | 13 |
| Necrotising fasciitis | 12 |
| Klebsiella infection | 12 |
| Erysipelas | 11 |
| Infection parasitic | 11 |
| Otitis externa | 11 |
| Pneumonia streptococcal | 11 |
| Large intestine infection | 11 |
| Epididymitis | 10 |
| Laryngitis | 10 |
| Liver abscess | 10 |
| Meningitis viral | 10 |
| Tracheobronchitis | 10 |
| Vaginal infection | 10 |
| Oral infection | 10 |
| Helicobacter infection | 10 |
| Arthritis infective | 10 |
| Pilonidal disease | 10 |
| Diarrhoea infectious | 9 |
| Escherichia sepsis | 9 |
| Hepatitis A | 9 |
| Infection susceptibility increased | 9 |
| Meningitis bacterial | 9 |
| Pulmonary tuberculosis | 9 |
| Pyelonephritis acute | 9 |
| Staphylococcal bacteraemia | 9 |
| Escherichia urinary tract infection | 9 |
| Bronchitis viral | 9 |
| Gastroenteritis norovirus | 9 |
| Complicated appendicitis | 9 |
| Folliculitis | 8 |
| Pneumonia pneumococcal | 8 |
| Pneumonia staphylococcal | 8 |
| Vulvovaginal candidiasis | 8 |
| Streptococcal sepsis | 8 |
| Gastric infection | 8 |
| Biliary sepsis | 8 |
| Infected cyst | 8 |
| Enterococcal infection | 8 |
| Lower respiratory tract infection viral | 8 |
| Oral herpes | 8 |
| Bronchiolitis | 7 |
| Chronic sinusitis | 7 |
| Encephalitis viral | 7 |
| Fungal skin infection | 7 |
| Lyme disease | 7 |
| Oesophageal candidiasis | 7 |
| Pneumonia mycoplasmal | 7 |
| Viral pericarditis | 7 |
| Pneumonia necrotising | 7 |
| Catheter site infection | 7 |
| Acarodermatitis | 7 |
| Asymptomatic COVID-19 | 7 |
| Disseminated tuberculosis | 6 |
| Labyrinthitis | 6 |
| Mastitis | 6 |
| Pneumonia klebsiella | 6 |
| Rectal abscess | 6 |
| Appendiceal abscess | 6 |
| Abscess neck | 6 |
| Bacterial sepsis | 6 |
| Escherichia bacteraemia | 6 |
| Alpha haemolytic streptococcal infection | 6 |
| Enteritis infectious | 6 |
| Bronchitis bacterial | 6 |
| Post procedural sepsis | 6 |
| Pneumocystis jirovecii pneumonia | 6 |
| Abscess intestinal | 5 |
| Acquired immunodeficiency syndrome | 5 |
| Herpes virus infection | 5 |
| Impetigo | 5 |
| Infected skin ulcer | 5 |
| Intestinal gangrene | 5 |
| Lymphangitis | 5 |
| Mononucleosis syndrome | 5 |
| Nosocomial infection | 5 |
| Onychomycosis | 5 |
| Paronychia | 5 |
| Pelvic inflammatory disease | 5 |
| Periodontitis | 5 |
| Pneumonia pseudomonal | 5 |
| Subacute endocarditis | 5 |
| Campylobacter infection | 5 |
| Cardiac valve vegetation | 5 |
| Clostridium colitis | 5 |
| Wound infection staphylococcal | 5 |
| Diabetic foot infection | 5 |

| **Supplementary Table 2: Terms of infections included in the analysis** |
| --- |
| Bacterial disease carrier |
| Diphtheria carrier |
| HIV carrier |
| Sexually transmitted disease carrier |
| Typhoid carrier |
| Viral hepatitis carrier |
| Infectious disease carrier |
| HTLV-1 carrier |
| Mycobacterial disease carrier |
| Fungal disease carrier |
| Viral disease carrier |
| SARS-CoV-2 carrier |
| Arthritis reactive |
| Chronic gastritis |
| Chronic hepatitis |
| Encephalitis post immunisation |
| Encephalitis post varicella |
| Endocarditis rheumatic |
| Erythema marginatum |
| Guillain-Barre syndrome |
| Hepatitis chronic active |
| Hepatitis chronic persistent |
| Hepatitis fulminant |
| Kawasaki's disease |
| Keratoderma blenorrhagica |
| Pericarditis rheumatic |
| Post polio syndrome |
| Post streptococcal glomerulonephritis |
| Post herpetic neuralgia |
| Progressive massive fibrosis |
| Psorospermiasis |
| Reye's syndrome |
| Rheumatic fever |
| Roseola |
| Secondary amyloidosis |
| Stevens-Johnson syndrome |
| Subacute sclerosing panencephalitis |
| Sydenham's chorea |
| Thyroiditis subacute |
| Toxic epidermal necrolysis |
| Scleroedema |
| Malacoplakia vesicae |
| Rheumatic heart disease |
| Myocarditis post infection |
| Malacoplakia gastrointestinal |
| Poncet's disease |
| Post infection glomerulonephritis |
| Acute motor-sensory axonal neuropathy |
| Acute motor axonal neuropathy |
| Acute encephalitis with refractory, repetitive partial seizures |
| Bickerstaff's encephalitis |
| Sporadic infantile bilateral striatal necrosis |
| Granulomatous lymphadenitis |
| Febrile infection-related epilepsy syndrome |
| Malacoplakia |
| Cutaneous malacoplakia |
| Malacoplakia of bone |
| Subacute inflammatory demyelinating polyneuropathy |
| Degenerative multivalvular disease |
| SJS-TEN overlap |
| Infantile acropustulosis |
| Ciliary ganglionitis |
| Follicular cystitis |
| Bullous oedema of the bladder |
| Axonal and demyelinating polyneuropathy |
| Multisystem inflammatory syndrome in children |
| Multisystem inflammatory syndrome in adults |
| Multisystem inflammatory syndrome |
| Paediatric acute-onset neuropsychiatric syndrome |
| Intrauterine gas |
| Ascending flaccid paralysis |
| Air-borne transmission |
| Direct infection transmission |
| Faecal-oral transmission of infection |
| Food poisoning |
| Indirect infection transmission |
| Infection transmission via personal contact |
| Infection via vaccinee |
| Nosocomial infection |
| Sexual transmission of infection |
| Sexually transmitted disease |
| Vector-borne transmission of infection |
| Vertical infection transmission |
| Secondary transmission |
| Primary transmission |
| Human immunodeficiency virus transmission |
| Arthropod-borne disease |
| Iatrogenic infection |
| Community acquired infection |
| Vaccine virus shedding |
| Transmission of an infectious agent via product |
| Suspected transmission of an infectious agent via product |
| Tick paralysis |
| Transmission of an infectious agent via transplant |
| Vaccine bacteria shedding |
| Bloodborne infection |
| Actinomycosis |
| Actinomycotic abdominal infection |
| Actinomycotic pulmonary infection |
| Actinomycotic skin infection |
| Actinomycotic sepsis |
| Gastroenteritis aerobacter |
| Enterobacter infection |
| Enterobacter pneumonia |
| Enterobacter sepsis |
| Enterobacter tracheobronchitis |
| Enterobacter bacteraemia |
| Gastroenteritis aeromonas |
| Aeromonas infection |
| Anthrax |
| Cutaneous anthrax |
| Gastroenteritis bacillus |
| Gastrointestinal anthrax |
| Pneumonia anthrax |
| Anthrax sepsis |
| Bacillus infection |
| Bacillus bacteraemia |
| Shewanella algae bacteraemia |
| Abscess bacterial |
| Angina gangrenous |
| Anorectal cellulitis |
| Arteriosclerotic gangrene |
| Bacterial abscess central nervous system |
| Bacterial diarrhoea |
| Bacterial food poisoning |
| Bacterial pericarditis |
| Bacterial vaginosis |
| Bacteriuria |
| Bacteriuria in pregnancy |
| Breast cellulitis |
| Cellulitis |
| Cellulitis gangrenous |
| Cellulitis orbital |
| Colon gangrene |
| Diabetic gangrene |
| Diaphragmatic hernia gangrenous |
| Endocarditis bacterial |
| External ear cellulitis |
| Eye infection bacterial |
| Femoral hernia gangrenous |
| Folliculitis |
| Gangrene |
| Gangrene neonatal |
| Gastroenteritis paracolon bacillus |
| Gastrointestinal gangrene |
| Ileal gangrene |
| Incisional hernia gangrenous |
| Inguinal hernia gangrenous |
| Intestinal gangrene |
| Jejunal gangrene |
| Lacrimal sac cellulitis |
| Meningitis bacterial |
| Osler's nodes |
| Overgrowth bacterial |
| Paronychia |
| Pyomyositis |
| Scrotal gangrene |
| Small intestine gangrene |
| Sycosis barbae |
| Umbilical hernia gangrenous |
| Vulval cellulitis |
| Whipple's disease |
| Injection site cellulitis |
| Meningoencephalitis bacterial |
| Eczema impetiginous |
| Acinetobacter infection |
| Citrobacter infection |
| Propionibacterium infection |
| Empedobacter brevis infection |
| Vaginal cellulitis |
| Bacterial allergy |
| Antibiotic associated colitis |
| Tuberculous abscess central nervous system |
| Skin bacterial infection |
| Post procedural cellulitis |
| Catheter site cellulitis |
| Arthritis bacterial |
| Bacterial sepsis |
| Urinary tract infection bacterial |
| Micrococcus infection |
| Peptostreptococcus infection |
| Stenotrophomonas sepsis |
| Stenotrophomonas infection |
| Veillonella infection |
| Micrococcal sepsis |
| Acid fast bacilli infection |
| Citrobacter sepsis |
| Flavobacterium infection |
| Bifidobacterium infection |
| Morganella infection |
| Asymptomatic bacteriuria |
| Bacterial dacryocystitis |
| Ovarian bacterial infection |
| Periorbital cellulitis |
| Upper respiratory tract infection bacterial |
| Pharyngitis bacterial |
| Bacterial labyrinthitis |
| Bacteroides infection |
| Bacterial pyelonephritis |
| Respiratory tract infection bacterial |
| Sinusitis bacterial |
| Abdominal hernia gangrenous |
| Bacterial infection |
| Pneumonia bacterial |
| Anorectal infection bacterial |
| Spontaneous bacterial peritonitis |
| Gastroenteritis bacterial |
| Genital infection bacterial |
| Hernia gangrenous |
| Oesophagitis bacterial |
| Zoonotic bacterial infection |
| Bronchitis bacterial |
| Conjunctivitis bacterial |
| Gastritis bacterial |
| Keratitis bacterial |
| Lactobacillus infection |
| Perichondritis |
| Peritonitis bacterial |
| Laryngitis bacterial |
| Bacterial iritis |
| Implant site cellulitis |
| Lower respiratory tract infection bacterial |
| Incision site cellulitis |
| Cellulitis of male external genital organ |
| Acinetobacter bacteraemia |
| Rhodococcus infection |
| Otitis media bacterial |
| Otitis externa bacterial |
| Bacterial rhinitis |
| Pancreatitis bacterial |
| Splenic infection bacterial |
| Cystitis bacterial |
| Biliary tract infection bacterial |
| Enterocolitis bacterial |
| Mastitis bacterial |
| Hepatic infection bacterial |
| Myocarditis bacterial |
| Lymphadenitis bacterial |
| Oral bacterial infection |
| Tonsillitis bacterial |
| Osteomyelitis bacterial |
| Wound infection bacterial |
| Nail bed infection bacterial |
| Application site cellulitis |
| Infusion site cellulitis |
| Urogenital infection bacterial |
| Bacterial tracheitis |
| Superinfection bacterial |
| Ear infection bacterial |
| Proctitis bacterial |
| Pleural infection bacterial |
| Gastrointestinal bacterial infection |
| Stomatococcal infection |
| Bacterascites |
| Gangrenous balanitis |
| Alcaligenes infection |
| Eubacterium infection |
| Vaccination site cellulitis |
| Sphingomonas paucimobilis infection |
| Cronobacter bacteraemia |
| Cronobacter infection |
| Meningitis cronobacter |
| Bacterial prostatitis |
| Cronobacter necrotising enterocolitis |
| Pantoea agglomerans infection |
| Raoultella ornithinolytica infection |
| Leptotrichia infection |
| Leuconostoc infection |
| Methylobacterium infection |
| Botryomycosis |
| Endometritis bacterial |
| Stoma site cellulitis |
| Administration site cellulitis |
| Medical device site cellulitis |
| Bacterial colitis |
| Janeway lesion |
| Vessel puncture site cellulitis |
| Bacterial parotitis |
| Granulicatella bacteraemia |
| Granulicatella infection |
| Parvimonas infection |
| Gastrointestinal bacterial overgrowth |
| Brevibacterium infection |
| Systemic bacterial infection |
| Bacterial blepharitis |
| Puncture site cellulitis |
| Parvimonas micra infection |
| Bacterial abdominal infection |
| Bacterial salpingitis |
| Bacterial urethritis |
| Bacterial ureteritis |
| Purple urine bag syndrome |
| Bacterial vulvovaginitis |
| Pneumonia acinetobacter |
| Citrobacter bacteraemia |
| Tracheobronchitis bacterial |
| Delftia acidovorans infection |
| Sphingomonas paucimobilis bacteraemia |
| Nephritis bacterial |
| Providencia urinary tract infection |
| Providencia infection |
| Achromobacter infection |
| Acinetobacter sepsis |
| Aerococcus urinae infection |
| Bacterial endophthalmitis |
| Stenotrophomonas bacteraemia |
| Weissella infection |
| Bacterial myositis |
| Bacterial gingivitis |
| Penile gangrene |
| Abiotrophia defectiva endocarditis |
| Pleurisy bacterial |
| Leclercia bacteraemia |
| Encephalomyelitis bacterial |
| Folliculitis genital |
| Neonatal bacterial pneumonia |
| Pertussis |
| Pneumonia bordetella |
| Bordetella infection |
| Acrodermatitis chronica atrophicans |
| Lyme disease |
| Relapsing fever |
| Meningitis borrelia |
| Neuroborreliosis |
| Borrelia infection |
| Erythema migrans |
| Lyme carditis |
| Post treatment Lyme disease syndrome |
| Brucellosis |
| Brucella sepsis |
| Capnocytophaga infection |
| Capnocytophaga sepsis |
| Botulism |
| Clostridium difficile colitis |
| Enteritis necroticans |
| Gas gangrene |
| Gastroenteritis clostridial |
| Pseudomembranous colitis |
| Tetanus |
| Tetanus neonatorum |
| Clostridium difficile infection |
| Clostridium colitis |
| Clostridium bacteraemia |
| Clostridial infection |
| Hepatic gas gangrene |
| Clostridial sepsis |
| Obstetrical tetanus |
| Diphtheria |
| Erythrasma |
| Corynebacterium sepsis |
| Corynebacterium infection |
| Corynebacterium bacteraemia |
| Cellulitis enterococcal |
| Endocarditis enterococcal |
| Enterococcal bacteraemia |
| Meningitis enterococcal |
| Urinary tract infection enterococcal |
| Enterococcal sepsis |
| Enterococcal infection |
| Enterococcal gastroenteritis |
| Erysipelothrix infection |
| Erysipelothrix sepsis |
| Cystitis escherichia |
| Escherichia sepsis |
| Gastroenteritis Escherichia coli |
| Pneumonia escherichia |
| Escherichia urinary tract infection |
| Escherichia bacteraemia |
| Escherichia vaginitis |
| Escherichia infection |
| Prostatitis Escherichia coli |
| Escherichia pyelonephritis |
| Escherichia peritonitis |
| Meningitis Escherichia |
| Pneumonia tularaemia |
| Tularaemia |
| Necrobacillosis |
| Fusobacterium infection |
| Necrotising ulcerative gingivostomatitis |
| Necrotising ulcerative periodontitis |
| Lemierre syndrome |
| Lineal gingival erythema |
| Gardnerella infection |
| Vaginitis gardnerella |
| Bronchitis haemophilus |
| Chancroid |
| Endocarditis haemophilus |
| Epiglottitis haemophilus |
| Meningitis haemophilus |
| Pneumonia haemophilus |
| Septic arthritis haemophilus |
| Haemophilus sepsis |
| Haemophilus bacteraemia |
| Haemophilus infection |
| Otitis media haemophilus |
| Brazilian purpuric fever |
| Aggregatibacter infection |
| Gastric ulcer helicobacter |
| Helicobacter infection |
| Helicobacter sepsis |
| Helicobacter gastritis |
| Peptic ulcer helicobacter |
| Helicobacter duodenitis |
| Helicobacter duodenal ulcer |
| Cystitis klebsiella |
| Granuloma inguinale |
| Pneumonia klebsiella |
| Rhinoscleroma |
| Klebsiella sepsis |
| Klebsiella bacteraemia |
| Klebsiella infection |
| Klebsiella pneumoniae invasive syndrome |
| Pneumonia legionella |
| Pontiac fever |
| Legionella infection |
| Anicteric leptospirosis |
| Leptospirosis |
| Meningitis leptospiral |
| Weil's disease |
| Leptospira sepsis |
| Listeriosis |
| Meningitis listeria |
| Listeria encephalitis |
| Listeria sepsis |
| Cutaneous listeriosis |
| Listeraemia |
| Gastroenteritis listeria |
| Otitis media moraxella |
| Pneumonia moraxella |
| Moraxella infection |
| Bronchitis moraxella |
| Arthritis gonococcal |
| Cervicitis gonococcal |
| Conjunctivitis gonococcal neonatal |
| Cystitis gonococcal |
| Encephalitis meningococcal |
| Endocarditis gonococcal |
| Endocarditis meningococcal |
| Endometritis gonococcal |
| Epididymo-orchitis gonococcal |
| Eye infection gonococcal |
| Gonorrhoea |
| Keratosis gonococcal |
| Meningitis gonococcal |
| Meningitis meningococcal |
| Meningococcal carditis |
| Meningococcal infection |
| Meningococcal sepsis |
| Myocarditis meningococcal |
| Optic neuritis meningococcal |
| Pericarditis gonococcal |
| Pericarditis meningococcal |
| Perihepatitis gonococcal |
| Peritonitis gonococcal |
| Proctitis gonococcal |
| Prostatitis gonococcal |
| Salpingitis gonococcal |
| Seminal vesiculitis gonococcal |
| Septic arthritis neisserial |
| Urethritis gonococcal |
| Waterhouse-Friderichsen syndrome |
| Meningococcal bacteraemia |
| Genitourinary tract gonococcal infection |
| Neisseria infection |
| Oropharyngeal gonococcal infection |
| Vulvovaginitis gonococcal |
| Gonococcal pelvic inflammatory disease |
| Gonococcal heart disease |
| Gonococcal infection |
| Anal gonococcal infection |
| Nocardiosis |
| Nocardia sepsis |
| Pulmonary nocardiosis |
| Cutaneous nocardiosis |
| Cerebral nocardiosis |
| Cellulitis pasteurella |
| Pasteurella infection |
| Sepsis pasteurella |
| Porphyromonas infection |
| Porphyromonas bacteraemia |
| Gastroenteritis proteus |
| Proteus infection |
| Pneumonia proteus |
| Cystitis pseudomonal |
| Gastroenteritis pseudomonas |
| Pneumonia pseudomonal |
| Pseudomonal sepsis |
| Pseudomonal bacteraemia |
| Wound infection pseudomonas |
| Pseudomonas infection |
| Urinary tract infection pseudomonal |
| Pseudomonas bronchitis |
| Endocarditis pseudomonal |
| Pseudomonas aeruginosa meningitis |
| Pseudomonas peritonitis |
| Pseudomonal skin infection |
| Green nail syndrome |
| Arthritis salmonella |
| Gastroenteritis salmonella |
| Meningitis salmonella |
| Osteomyelitis salmonella |
| Paratyphoid fever |
| Pneumonia salmonella |
| Salmonellosis |
| Typhoid fever |
| Salmonella sepsis |
| Salmonella bacteraemia |
| Aortitis salmonella |
| Serratia sepsis |
| Serratia bacteraemia |
| Serratia infection |
| Pneumonia serratia |
| Gastroenteritis shigella |
| Shigella infection |
| Shigella sepsis |
| Spirillary fever |
| Bullous impetigo |
| Cellulitis staphylococcal |
| Endocarditis staphylococcal |
| Eye infection staphylococcal |
| Furuncle |
| Gastroenteritis staphylococcal |
| Meningitis staphylococcal |
| Necrotising fasciitis staphylococcal |
| Pneumonia staphylococcal |
| Septic arthritis staphylococcal |
| Staphylococcal abscess |
| Staphylococcal impetigo |
| Staphylococcal scalded skin syndrome |
| Staphylococcal toxaemia |
| Toxic shock syndrome staphylococcal |
| Bursitis infective staphylococcal |
| Staphylococcal bacteraemia |
| Staphylococcal sepsis |
| Ear infection staphylococcal |
| Staphylococcal infection |
| Wound infection staphylococcal |
| Staphylococcal pharyngitis |
| Urinary tract infection staphylococcal |
| Periporitis staphylogenes |
| Staphylococcal osteomyelitis |
| Staphylococcal skin infection |
| Staphylococcal mediastinitis |
| Staphylococcal parotitis |
| Staphylococcal blepharitis |
| Vulvovaginitis staphylococcal |
| Otitis media staphylococcal |
| Giant fornix syndrome |
| Cellulitis streptococcal |
| Erysipelas |
| Meningitis pneumococcal |
| Meningitis streptococcal |
| Necrotising fasciitis streptococcal |
| Peritonitis pneumococcal |
| Pharyngitis streptococcal |
| Pneumonia pneumococcal |
| Pneumonia streptococcal |
| Pyoderma streptococcal |
| Scarlet fever |
| Streptococcal abscess |
| Streptococcal impetigo |
| Tonsillitis streptococcal |
| Toxic shock syndrome streptococcal |
| Streptococcal sepsis |
| Streptococcal bacteraemia |
| Beta haemolytic streptococcal infection |
| Group B streptococcus neonatal sepsis |
| Pneumococcal sepsis |
| Alpha haemolytic streptococcal infection |
| Pneumococcal bacteraemia |
| Pneumococcal infection |
| Streptococcal infection |
| Bronchitis pneumococcal |
| Vulvovaginitis streptococcal |
| Cervicitis streptococcal |
| Septic arthritis streptococcal |
| Perianal streptococcal infection |
| Streptococcal urinary tract infection |
| Severe invasive streptococcal infection |
| Streptococcal endocarditis |
| Otitis media pneumococcal |
| Bullous erysipelas |
| Streptococcal bronchitis |
| Bacillary angiomatosis |
| Bartonellosis |
| Cat scratch disease |
| Trench fever |
| Systemic bartonellosis |
| Peruvian wart |
| Adenopathy syphilitic |
| Alopecia syphilitic |
| Aortic aneurysm syphilitic |
| Aortitis syphilitic |
| Cardiovascular syphilis |
| Cerebral aneurysm ruptured syphilitic |
| Congenital syphilis |
| Congenital syphilitic encephalitis |
| Congenital syphilitic meningitis |
| Endocarditis syphilitic |
| Eye infection syphilitic |
| Hepatitis syphilitic |
| Myocarditis syphilitic |
| Neurosyphilis |
| Pericarditis syphilitic |
| Peritonitis syphilitic |
| Pinta |
| Pulmonary syphilis |
| Renal syphilis |
| Syphilis anal |
| Syphilis genital |
| Syphilis musculoskeletal |
| Syphilitic endocarditis of heart valve |
| Yaws |
| Yaws of bone |
| Yaws of skin |
| Endemic syphilis |
| Congenital syphilitic osteochondritis |
| Malignant syphilis |
| Tertiary syphilis |
| Secondary syphilis |
| Primary syphilis |
| Spirochaetal infection |
| Syphilis |
| Condyloma latum |
| Latent syphilis |
| Brachyspira infection |
| Syphilitic pelvic inflammatory disease |
| Cholera |
| Gastroenteritis vibrio |
| Vibrio vulnificus infection |
| Bubonic plague |
| Gastroenteritis yersinia |
| Plague |
| Pneumonic plague |
| Yersinia bacteraemia |
| Plague sepsis |
| Yersinia infection |
| Yersinia sepsis |
| Yersinia meningitis |
| Septic arthritis streptobacillus |
| Streptobacillary fever |
| Streptobacillus infection |
| Campylobacter gastroenteritis |
| Campylobacter infection |
| Campylobacter sepsis |
| Campylobacter colitis |
| Campylobacter urinary tract infection |
| Burkholderia cepacia complex infection |
| Burkholderia cepacia complex sepsis |
| Burkholderia mallei infection |
| Burkholderia pseudomallei infection |
| Burkholderia gladioli infection |
| Burkholderia infection |
| Anal chlamydia infection |
| Chlamydial pelvic inflammatory disease |
| Conjunctivitis chlamydial |
| Eye infection chlamydial |
| Genitourinary chlamydia infection |
| Inclusion conjunctivitis |
| Lymphogranuloma venereum |
| Peritoneal chlamydia infection |
| Pharyngeal chlamydia infection |
| Pneumonia chlamydial |
| Proctitis chlamydial |
| Psittacosis |
| Trachoma |
| Urethritis chlamydial |
| Gynaecological chlamydia infection |
| Inclusion conjunctivitis neonatal |
| Chlamydial infection |
| Chlamydial cervicitis |
| Vaginitis chlamydial |
| Vulvovaginitis chlamydial |
| Respiratory tract chlamydial infection |
| Acariasis |
| Bed bug infestation |
| Flea infestation |
| Hirudiniasis |
| Lice infestation |
| Myiasis |
| Arthropod infestation |
| Infestation |
| Acarodermatitis |
| Demodicidosis |
| Tungiasis |
| Trombidiasis |
| Allescheriosis |
| Aspergilloma |
| Aspergillosis oral |
| Bronchopulmonary aspergillosis |
| Bronchopulmonary aspergillosis allergic |
| Sinusitis aspergillus |
| Cerebral aspergillosis |
| Oro-pharyngeal aspergillosis |
| Meningitis aspergillus |
| Aspergillus infection |
| Disseminated aspergillosis |
| Blastomycosis |
| Epididymitis blastomyces |
| Osteomyelitis blastomyces |
| Cutaneous blastomycosis |
| Disseminated blastomycosis |
| Pulmonary blastomycosis |
| Anal candidiasis |
| Balanitis candida |
| Candida nappy rash |
| Endocarditis candida |
| Gastrointestinal candidiasis |
| Genital candidiasis |
| Meningitis candida |
| Mucocutaneous candidiasis |
| Nail candida |
| Neonatal candida infection |
| Oesophageal candidiasis |
| Oral candidiasis |
| Otitis externa candida |
| Proctitis monilial |
| Respiratory moniliasis |
| Systemic candida |
| Vulvovaginal candidiasis |
| Hepatic candidiasis |
| Nasal candidiasis |
| Oropharyngeal candidiasis |
| Hepatosplenic candidiasis |
| Splenic candidiasis |
| Candida pneumonia |
| Candida sepsis |
| Skin candida |
| Peritoneal candidiasis |
| Bladder candidiasis |
| Stoma site candida |
| Candida endophthalmitis |
| Candidiasis of trachea |
| Candida osteomyelitis |
| Candida retinitis |
| Candida cervicitis |
| Candida infection |
| Cerebral candidiasis |
| Denture stomatitis |
| Candida urethritis |
| Urinary tract candidiasis |
| Chromoblastomycosis |
| Coccidioidomycosis |
| Meningitis coccidioides |
| Coccidioides encephalitis |
| Cutaneous coccidioidomycosis |
| Disseminated coccidioidomycosis |
| Gastroenteritis cryptococcal |
| Cryptococcosis |
| Disseminated cryptococcosis |
| Meningitis cryptococcal |
| Cryptococcal cutaneous infection |
| Cryptococcal fungaemia |
| Pneumonia cryptococcal |
| Neurocryptococcosis |
| Osseous cryptococcosis |
| Laryngeal cryptococcosis |
| Cryptococcal meningoencephalitis |
| Eye infection fungal |
| Fungal abscess central nervous system |
| Fungal cystitis |
| Fungal endocarditis |
| Fungal infection |
| Fungal paronychia |
| Fungal skin infection |
| Lobomycosis |
| Meningitis fungal |
| Mucormycosis |
| Mycetoma mycotic |
| Mycotic corneal ulcer |
| Mycotoxicosis |
| Onychomycosis |
| Urinary tract infection fungal |
| Gastrointestinal fungal infection |
| Fungal oesophagitis |
| Cerebral fungal infection |
| Fusarium infection |
| Systemic mycosis |
| Otitis externa fungal |
| Necrotising fasciitis fungal |
| Torulopsis infection |
| Trichosporon infection |
| Alternaria infection |
| Geotrichum infection |
| Sinusitis fungal |
| Fungal sepsis |
| Myocarditis mycotic |
| Scedosporium infection |
| Respiratory tract infection fungal |
| Arthritis fungal |
| Fungal peritonitis |
| Genital infection fungal |
| Oral fungal infection |
| Pneumonia fungal |
| Bronchitis fungal |
| Oropharyngitis fungal |
| Gastritis fungal |
| Upper respiratory fungal infection |
| Keratitis fungal |
| Proctitis fungal |
| Mycotic endophthalmitis |
| Vulvovaginal mycotic infection |
| Encephalitis fungal |
| Fungal labyrinthitis |
| Otitis media fungal |
| Fungal rhinitis |
| Lower respiratory tract infection fungal |
| Pancreatitis fungal |
| Splenic infection fungal |
| Biliary tract infection fungal |
| Enterocolitis fungal |
| Lymphadenitis fungal |
| Mastitis fungal |
| Pyelonephritis fungal |
| Hepatic infection fungal |
| Pericarditis fungal |
| Tonsillitis fungal |
| Osteomyelitis fungal |
| Wound infection fungal |
| Abscess fungal |
| Urogenital infection fungal |
| Superinfection fungal |
| Laryngitis fungal |
| Fungal foot infection |
| Pulmonary trichosporonosis |
| Disseminated trichosporonosis |
| Anal fungal infection |
| Fungal retinitis |
| Ear infection fungal |
| Fungal tracheitis |
| Central nervous system fungal infection |
| Disseminated mucormycosis |
| Pythium insidiosum infection |
| Tongue fungal infection |
| Overgrowth fungal |
| Fungal pharyngitis |
| Rhinocerebral mucormycosis |
| Funguria |
| Severe asthma with fungal sensitisation |
| Pulmonary mucormycosis |
| Penicillium infection |
| Fungal balanitis |
| Scopulariopsis infection |
| Neoscytalidium infection |
| Phaeohyphomycosis |
| Phaeohyphomycotic brain abscess |
| Conjunctivitis fungal |
| Fungal urethritis |
| Aureobasidium pullulans infection |
| Gastrointestinal mucormycosis |
| Cutaneous mucormycosis |
| Allergic bronchopulmonary mycosis |
| Mastoiditis fungal |
| Fungal myositis |
| Acute pulmonary histoplasmosis |
| Chronic pulmonary histoplasmosis |
| Endocarditis histoplasma |
| Histoplasmosis |
| Histoplasmosis disseminated |
| Meningitis histoplasma |
| Pericarditis histoplasma |
| Retinitis histoplasma |
| Histoplasmosis cutaneous |
| Presumed ocular histoplasmosis syndrome |
| Pulmonary histoplasmosis |
| Paracoccidioides infection |
| Pulmonary paracoccidioidomycosis |
| Disseminated paracoccidioidomycosis |
| Pneumocystis jirovecii pneumonia |
| Pneumocystis jirovecii infection |
| Pseudallescheria sepsis |
| Pseudallescheria infection |
| Rhinosporidiosis |
| Cutaneous sporotrichosis |
| Sporotrichosis |
| Pulmonary sporotrichosis |
| Disseminated sporotrichosis |
| Osteoarticular sporotrichosis |
| Anal tinea |
| Black piedra |
| Body tinea |
| Dermatophytosis |
| Dermatophytosis of nail |
| Kerion |
| Tinea blanca |
| Tinea capitis |
| Tinea cruris |
| Tinea nigra |
| Tinea pedis |
| Tinea imbricata |
| Trichophytic granuloma |
| Malassezia infection |
| Microsporum infection |
| Tinea versicolour |
| Tinea barbae |
| Tinea infection |
| Tinea manuum |
| Trichophytosis |
| Tinea faciei |
| Exserohilum infection |
| Meningitis exserohilum |
| Diphyllobothriasis |
| Echinococciasis |
| Hepatic echinococciasis |
| Hymenolepiasis |
| Pulmonary echinococciasis |
| Taeniasis |
| Thyroid echinococciasis |
| Neurocysticercosis |
| Cestode infection |
| Renal echinococciasis |
| Dipylidiasis |
| Echinococciasis of bone |
| Ophthalmic cysticercosis |
| Arthritis helminthic |
| Helminthic infection |
| Meningoencephalitis helminthic |
| Skin infection helminthic |
| Eye infection helminthic |
| Upper respiratory tract infection helminthic |
| Pancreatitis helminthic |
| Splenic infection helminthic |
| Cystitis helminthic |
| Biliary tract infection helminthic |
| Enterocolitis helminthic |
| Gastritis helminthic |
| Lymphadenitis helminthic |
| Hepatic infection helminthic |
| Myocarditis helminthic |
| Pericarditis helminthic |
| Genital infection helminthic |
| Wound infection helminthic |
| Pneumonia helminthic |
| Peritonitis helminthic |
| Endocarditis helminthic |
| Vulvovaginitis helminthic |
| Oral helminthic infection |
| Anisakiasis |
| Ascariasis |
| Capillariasis |
| Dracunculiasis |
| Enterobiasis |
| Filariasis |
| Filariasis lymphatic |
| Gnathostomiasis |
| Hookworm infection |
| Onchocerciasis |
| Strongyloidiasis |
| Toxocariasis |
| Trichiniasis |
| Trichostrongyliasis |
| Trichuriasis |
| Cutaneous larva migrans |
| Nematodiasis |
| Tropical eosinophilia |
| Angiostrongylus infection |
| Dirofilariasis |
| Syngamiasis |
| Disseminated strongyloidiasis |
| Onchodermatitis |
| Clonorchiasis |
| Fascioliasis |
| Fasciolopsiasis |
| Heterophyiasis |
| Metagonimiasis |
| Opisthorchiasis |
| Paragonimiasis |
| Schistosomiasis |
| Schistosomiasis bladder |
| Schistosomiasis cutaneous |
| Schistosomiasis liver |
| Trematode infection |
| Dicrocoeliasis |
| Infected bunion |
| Osteomyelitis |
| Osteomyelitis acute |
| Osteomyelitis chronic |
| Purulent synovitis |
| Abscess jaw |
| Infective chondritis |
| Joint abscess |
| Intervertebral discitis |
| Arthritis infective |
| Bursitis infective |
| Infective spondylitis |
| Sternitis |
| Bone abscess |
| Paraspinal abscess |
| Injection site joint infection |
| Infective periostitis |
| Subperiosteal abscess |
| Administration site joint infection |
| Application site joint infection |
| Infusion site joint infection |
| Medical device site joint infection |
| Vaccination site joint infection |
| Fracture infection |
| Infected gouty tophus |
| Breast abscess |
| Breast discharge infected |
| Mastitis |
| Mastitis postpartum |
| Nipple infection |
| Recurrent subareolar breast abscess |
| Endocarditis |
| Myocarditis septic |
| Prosthetic valve endocarditis |
| Subacute endocarditis |
| Acute endocarditis |
| Purulent pericarditis |
| Cardiac infection |
| Cardiac valve vegetation |
| Myocardiac abscess |
| Pericarditis infective |
| Cardiac valve abscess |
| Myocarditis infectious |
| Infective pericardial effusion |
| Septic endocarditis |
| Septic cardiomyopathy |
| Brain abscess |
| Cavernous sinus thrombosis |
| CNS ventriculitis |
| Dural abscess |
| Encephalitis |
| Encephalomyelitis |
| Ependymitis |
| Meningitis |
| Meningitis aseptic |
| Myelitis |
| Encephalitis brain stem |
| Spinal cord infection |
| Neurological infection |
| Encephalitis lethargica |
| Panencephalitis |
| Meningitis neonatal |
| Central nervous system infection |
| Central nervous system abscess |
| Extradural abscess |
| Cranial nerve infection |
| Subarachnoid abscess |
| Brain empyema |
| Spinal empyema |
| Spinal cord abscess |
| Intracranial infection |
| Subdural abscess |
| Abscess of salivary gland |
| Abscess oral |
| Gingivitis |
| Parotid abscess |
| Parotitis |
| Pericoronitis |
| Periodontal destruction |
| Periodontitis |
| Pulpitis dental |
| Sialoadenitis |
| Tooth abscess |
| Oral infection |
| Tooth infection |
| Ludwig angina |
| Gingival abscess |
| Oral pustule |
| Dental gangrene |
| Dental fistula |
| Lip infection |
| Tongue abscess |
| Alveolar osteitis |
| Infective glossitis |
| Root canal infection |
| Peri-implantitis |
| Papillon-Lefevre syndrome |
| Submandibular abscess |
| Abscess of external auditory meatus |
| Ear infection |
| Ear lobe infection |
| Labyrinthitis |
| Mastoiditis |
| Otitis externa |
| Otitis media |
| Otitis media acute |
| Otitis media chronic |
| Otosalpingitis |
| Petrositis |
| Mastoid empyema |
| Mastoid abscess |
| Myringitis |
| Gradenigo's syndrome |
| Infected aural fistula |
| Abscess of eyelid |
| Chorioretinitis |
| Conjunctivitis |
| Corneal abscess |
| Dacryocanaliculitis |
| Dacryocystitis |
| Endophthalmitis |
| Eye abscess |
| Eye infection |
| Eyelid boil |
| Eyelid infection |
| Hordeolum |
| Hypopyon |
| Ophthalmia neonatorum |
| Retinitis |
| Vitreous abscess |
| Periorbital infection |
| Periorbital abscess |
| Eye infection intraocular |
| Infectious iridocyclitis |
| Infectious crystalline keratopathy |
| Corneal infection |
| Orbital infection |
| Blebitis |
| Infective episcleritis |
| Infective iritis |
| Infective uveitis |
| Oculoglandular syndrome |
| Infective corneal ulcer |
| Infective keratitis |
| Infective scleritis |
| Keratouveitis |
| Lacrimal gland abscess |
| Vitritis infective |
| Abdominal wall abscess |
| Abscess intestinal |
| Appendicitis |
| Appendicitis perforated |
| Colostomy infection |
| Diarrhoea infectious |
| Diarrhoea infectious neonatal |
| Diverticulitis |
| Diverticulitis intestinal haemorrhagic |
| Gastroenteritis |
| Gastrointestinal infection |
| Peritoneal abscess |
| Peritonitis |
| Retroperitoneal abscess |
| Anal abscess |
| Rectal abscess |
| Pancreatic abscess |
| Douglas' abscess |
| Appendiceal abscess |
| Retroperitoneal infection |
| Intestinal fistula infection |
| Abdominal wall infection |
| Dysentery |
| Anal fistula infection |
| Haemorrhoid infection |
| Pyloric abscess |
| Pancreas infection |
| Perirectal abscess |
| Subdiaphragmatic abscess |
| Abdominal infection |
| Gastric infection |
| Oesophageal infection |
| Enterocolitis infectious |
| Enteritis infectious |
| Abdominal abscess |
| Anal infection |
| Anorectal infection |
| Perihepatic abscess |
| Parasitic gastroenteritis |
| Mesenteric abscess |
| Colonic abscess |
| Infective mesenteric panniculitis |
| Abdominal hernia infection |
| Retroperitonitis |
| Anal papillitis |
| Complicated appendicitis |
| Large intestine infection |
| Oesophageal abscess |
| Biloma infected |
| Diverticulitis intestinal perforated |
| Focal peritonitis |
| Infected large intestinal ulcer |
| Stump appendicitis |
| Abscess |
| Cross infection |
| Empyema |
| Infection |
| Infection in an immunocompromised host |
| Infection masked |
| Infection parasitic |
| Infection susceptibility increased |
| Injection site abscess |
| Injection site infection |
| Localised infection |
| Mediastinitis |
| Mononucleosis syndrome |
| Neonatal infective mastitis |
| Omphalitis |
| Opportunistic infection |
| Pathogen resistance |
| Pelvic abscess |
| Postoperative wound infection |
| Purulent discharge |
| Pyuria |
| Stitch abscess |
| Superinfection |
| Thymus abscess |
| Tick-borne fever |
| Toxic shock syndrome |
| Visceral larva migrans |
| Wound infection |
| Zoonosis |
| Genital infection |
| Infantile septic granulomatosis |
| Application site infection |
| Incision site abscess |
| Puncture site abscess |
| Lymph node abscess |
| Groin abscess |
| Abscess limb |
| Abscess soft tissue |
| Groin infection |
| Lymph gland infection |
| Postoperative abscess |
| Adrenal gland abscess |
| Transplant abscess |
| Thyroid gland abscess |
| Febrile infection |
| Thyroglossal cyst infection |
| Obstetric infection |
| Rectovaginal septum abscess |
| Catheter site infection |
| Parathyroid gland abscess |
| Graft infection |
| Vestibulitis |
| Parasitic oesophagitis |
| Application site abscess |
| Infected neoplasm |
| Infected cyst |
| Wound abscess |
| Pelvic infection |
| Stoma site abscess |
| Neutropenic infection |
| Implant site infection |
| Neonatal infection |
| Purulence |
| Adrenalitis |
| Genital abscess |
| Congenital infection |
| Respiratory tract infection |
| Parasite allergy |
| Puncture site infection |
| Implant site abscess |
| Stoma site infection |
| Device related infection |
| Abscess rupture |
| Virologic failure |
| Mucosal infection |
| Peripheral nerve infection |
| Administration site infection |
| Post procedural infection |
| Vaccine breakthrough infection |
| Administration site abscess |
| Vaccination site infection |
| Vaccination site abscess |
| Parasitic encephalitis |
| Tropical infectious disease |
| Infection reactivation |
| Infectious thyroiditis |
| Perihepatitis |
| Catheter site abscess |
| Infected fistula |
| Ear, nose and throat infection |
| Vessel puncture site infection |
| Instillation site abscess |
| Instillation site infection |
| Infected seroma |
| Medical device site abscess |
| Medical device site infection |
| Infected bite |
| Systemic infection |
| Vascular access site infection |
| Genital ulcer syndrome |
| Activated PI3 kinase delta syndrome |
| Anastomotic infection |
| Overgrowth of nonsusceptible organisms |
| Southern tick-associated rash illness |
| TORCH infection |
| Coinfection |
| Vascular access site cellulitis |
| Vascular access site abscess |
| Reproductive tract procedural infection |
| Infected metastasis |
| Atypical pneumonia |
| Bronchitis |
| Congenital pneumonia |
| Lower respiratory tract infection |
| Lung abscess |
| Mediastinal abscess |
| Pneumonia |
| Pneumonia aspiration |
| Tracheobronchitis |
| Sputum purulent |
| Neonatal pneumonia |
| Miliary pneumonia |
| Pneumonia necrotising |
| Infective exacerbation of chronic obstructive airways disease |
| Pyopneumothorax |
| Pleural infection |
| Young's syndrome |
| Embolic pneumonia |
| Post procedural pneumonia |
| Infective exacerbation of bronchiectasis |
| Infective pulmonary exacerbation of cystic fibrosis |
| Infectious pleural effusion |
| Haemorrhagic pneumonia |
| Parasitic pneumonia |
| Paracancerous pneumonia |
| Pulmonary gangrene |
| Abortion infected |
| Bartholin's abscess |
| Bartholinitis |
| Cervicitis |
| Endometritis |
| Endometritis decidual |
| Oophoritis |
| Ovarian abscess |
| Parametritis |
| Pelvic inflammatory disease |
| Puerperal pyrexia |
| Pyometra |
| Salpingitis |
| Umbilical sepsis |
| Vaginal infection |
| Vulval abscess |
| Vulvitis |
| Vulvovaginitis |
| Clitoris abscess |
| Vaginal abscess |
| Salpingo-oophoritis |
| Fallopian tube abscess |
| Myometritis |
| Induced abortion infection |
| Tubo-ovarian abscess |
| Intrauterine infection |
| Amniotic cavity infection |
| Post abortion infection |
| Genital infection female |
| Uterine infection |
| Parametric abscess |
| Uterine abscess |
| Funisitis |
| Lochial infection |
| Puerperal infection |
| Neovaginal infection |
| Pregnancy related infection |
| Epididymitis |
| Hydrocele male infected |
| Orchitis |
| Penile abscess |
| Prostatic abscess |
| Scrotal abscess |
| Prostate infection |
| Seminal vesicular infection |
| Penile infection |
| Scrotal infection |
| Genital infection male |
| Spermatic cord funiculitis |
| Testicular abscess |
| Balanoposthitis infective |
| Pyospermia |
| Seminal vesicle abscess |
| Scrotal cellulitis |
| Bacteraemia |
| Bacterial toxaemia |
| Endotoxic shock |
| Fungaemia |
| Postpartum sepsis |
| Sepsis |
| Sepsis neonatal |
| Septic embolus |
| Septic shock |
| Thrombophlebitis septic |
| Urosepsis |
| Neutropenic sepsis |
| Amniotic infection syndrome of Blane |
| Pulmonary sepsis |
| Septic necrosis |
| Sepsis syndrome |
| Septic phlebitis |
| Abdominal sepsis |
| Wound sepsis |
| Bacteroides bacteraemia |
| Viraemia |
| Pelvic sepsis |
| Septic rash |
| Post procedural sepsis |
| Endotoxaemia |
| Septic encephalopathy |
| Haematological infection |
| Device related sepsis |
| Bacterial translocation |
| Cerebral septic infarct |
| Septic vasculitis |
| Intestinal sepsis |
| Septic pulmonary embolism |
| Septic coagulopathy |
| Device related bacteraemia |
| Septic cerebral embolism |
| Neonatal bacteraemia |
| Abscess sweat gland |
| Acne pustular |
| Blister infected |
| Carbuncle |
| Dermatitis infected |
| Ecthyma |
| Eczema infected |
| Erysipeloid |
| Impetigo |
| Infected skin ulcer |
| Pustule |
| Pyoderma |
| Rash pustular |
| Skin graft infection |
| Skin infection |
| Subcutaneous abscess |
| Application site pustules |
| Infected naevus |
| Periumbilical abscess |
| Burn infection |
| Pilonidal cyst congenital |
| Injection site pustule |
| Sweat gland infection |
| Eyelid folliculitis |
| Application site folliculitis |
| Diabetic foot infection |
| Nail bed infection |
| Nail infection |
| Implant site pustules |
| Dermo-hypodermitis |
| Infusion site pustule |
| Vaccination site pustule |
| Tropical ulcer |
| Sebaceous gland infection |
| Pitted keratolysis |
| Infected dermal cyst |
| Instillation site pustules |
| Catheter site pustule |
| Administration site pustule |
| Medical device site pustule |
| Subgaleal abscess |
| Radiation site infection |
| Pilonidal disease |
| Dermal filler site infection |
| Acute sinusitis |
| Cellulitis laryngeal |
| Cellulitis pharyngeal |
| Chronic sinusitis |
| Chronic tonsillitis |
| Croup infectious |
| Epiglottitis |
| Epiglottitis obstructive |
| Laryngitis |
| Laryngotracheitis obstructive |
| Nasal abscess |
| Nasal vestibulitis |
| Nasopharyngitis |
| Peritonsillar abscess |
| Pharyngitis |
| Rhinitis |
| Sinusitis |
| Tonsillitis |
| Tracheitis |
| Tracheitis obstructive |
| Upper respiratory tract infection |
| Sinobronchitis |
| Pharyngotonsillitis |
| Pharyngolaryngeal abscess |
| Peritonsillitis |
| Adenoiditis |
| Rhinotracheitis |
| Rhinolaryngitis |
| Laryngopharyngitis |
| Upper aerodigestive tract infection |
| Tracheostomy infection |
| Pharyngeal abscess |
| Subglottic laryngitis |
| Tornwaldt bursitis |
| Tracheal abscess |
| Paranasal sinus abscess |
| Pharyngeal pustule |
| Parapharyngeal space infection |
| Paranasal mucopyocoele |
| Epiglottic abscess |
| Cystitis |
| Kidney infection |
| Perinephric abscess |
| Pyelitis |
| Pyelonephritis |
| Pyelonephritis acute |
| Pyelonephritis chronic |
| Pyonephrosis |
| Renal abscess |
| Urethral abscess |
| Urethral stricture post infection |
| Urethritis |
| Urinary tract infection |
| Urinary tract infection neonatal |
| Pyelocystitis |
| Ureteritis |
| Urinary bladder abscess |
| Urethral carbuncle |
| Emphysematous cystitis |
| Renal cyst infection |
| Genitourinary tract infection |
| Ureter abscess |
| Urinary tract abscess |
| Emphysematous pyelonephritis |
| Perinephritis |
| Bladder diverticulitis |
| Renal graft infection |
| Urethral discharge syndrome |
| Urinary meatitis |
| Infected urinoma |
| Urachal sinus infection |
| Fournier's gangrene |
| Infective myositis |
| Necrotising fasciitis |
| Muscle abscess |
| Chest wall abscess |
| Perineal abscess |
| Abscess neck |
| Fascial infection |
| Psoas abscess |
| Infective tenosynovitis |
| Soft tissue infection |
| Perineal infection |
| Necrotising soft tissue infection |
| Bezold abscess |
| Perineal cellulitis |
| Lymphangitis |
| Haematoma infection |
| Infusion site infection |
| Arteriovenous fistula site infection |
| Arteriovenous graft site infection |
| Phlebitis infective |
| Infected varicose vein |
| Infective thrombosis |
| Infective aneurysm |
| Shunt infection |
| Infusion site abscess |
| Infected lymphocele |
| Arteriovenous graft site abscess |
| Arteritis infective |
| Infective aortitis |
| Vascular device infection |
| Vascular graft infection |
| Pseudoaneurysm infection |
| Infected vasculitis |
| Hepatitis post transfusion |
| Liver abscess |
| Portal pyaemia |
| Splenic abscess |
| Gallbladder empyema |
| Hepatic cyst infection |
| Gallbladder abscess |
| Hepatic infection |
| Hepatobiliary infection |
| Emphysematous cholecystitis |
| Biliary sepsis |
| Biliary tract infection |
| Splenic infection |
| Cholecystitis infective |
| Biliary abscess |
| Cholangitis infective |
| Hepatosplenic abscess |
| Recurrent pyogenic cholangitis |
| Atypical mycobacterial lymphadenitis |
| Mycobacterium kansasii infection |
| Mycobacterium marinum infection |
| Mycobacterium fortuitum infection |
| Atypical mycobacterium pericarditis |
| Mycobacterium avium complex immune restoration disease |
| Mycobacterium avium complex infection |
| Atypical mycobacterial infection |
| Mycobacterial infection |
| Mycobacterium abscessus infection |
| Mycobacterium ulcerans infection |
| Disseminated mycobacterium avium complex infection |
| Atypical mycobacterial pneumonia |
| Mycobacterium chelonae infection |
| Mycobacterial peritonitis |
| Atypical mycobacterial lower respiratory tract infection |
| Superinfection mycobacterial |
| Mycobacterium haemophilum infection |
| Borderline leprosy |
| Indeterminate leprosy |
| Lepromatous leprosy |
| Leprosy |
| Tuberculoid leprosy |
| Type 1 lepra reaction |
| Type 2 lepra reaction |
| Adrenal gland tuberculosis |
| Bovine tuberculosis |
| Choroid tubercles |
| Congenital tuberculosis |
| Conjunctivitis tuberculous |
| Cutaneous tuberculosis |
| Disseminated tuberculosis |
| Ear tuberculosis |
| Epididymitis tuberculous |
| Erythema induratum |
| Lupus vulgaris |
| Lymph node tuberculosis |
| Meningitis tuberculous |
| Oesophageal tuberculosis |
| Pulmonary tuberculosis |
| Renal tuberculosis |
| Salpingitis tuberculous |
| Spleen tuberculosis |
| Thyroid tuberculosis |
| Tuberculosis |
| Tuberculosis bladder |
| Tuberculosis of eye |
| Tuberculosis of genitourinary system |
| Tuberculosis of intrathoracic lymph nodes |
| Tuberculosis of peripheral lymph nodes |
| Tuberculosis ureter |
| Tuberculous laryngitis |
| Tuberculous pleurisy |
| Tuberculoma of central nervous system |
| Peritoneal tuberculosis |
| Pericarditis tuberculous |
| Joint tuberculosis |
| Bone tuberculosis |
| Tuberculosis liver |
| Tuberculous tenosynovitis |
| Female genital tract tuberculosis |
| Male genital tract tuberculosis |
| Tuberculosis gastrointestinal |
| Tuberculosis of central nervous system |
| Extrapulmonary tuberculosis |
| Prostatitis tuberculous |
| Latent tuberculosis |
| Pulmonary tuberculoma |
| Silicotuberculosis |
| Tuberculous endometritis |
| Immune reconstitution inflammatory syndrome associated tuberculosis |
| Intestinal tuberculosis |
| Disseminated Bacillus Calmette-Guerin infection |
| Oral tuberculosis |
| Mammary tuberculosis |
| Tuberculosis of uterine cervix |
| Tuberculous pelvic inflammatory disease |
| Cardiac tuberculosis |
| Mycoplasmal postabortal fever |
| Mycoplasmal postpartum fever |
| Pelvic inflammatory disease mycoplasmal |
| Pericarditis mycoplasmal |
| Pharyngitis mycoplasmal |
| Pneumonia mycoplasmal |
| Pyelonephritis mycoplasmal |
| Tracheobronchitis mycoplasmal |
| Mycoplasma infection |
| Mycoplasma genitalium infection |
| Proctitis mycoplasmal |
| Cervicitis mycoplasmal |
| Urethritis mycoplasmal |
| Bronchitis mycoplasmal |
| Urethritis ureaplasmal |
| Epididymitis ureaplasmal |
| Ureaplasma infection |
| Ureaplasmal vulvovaginitis |
| Ureaplasma cervicitis |
| Amoebiasis |
| Amoebic brain abscess |
| Amoebic colitis |
| Amoebic dysentery |
| Amoebic lung abscess |
| Amoebic skin ulcer |
| Naegleria infection |
| Pericarditis amoebic |
| Acanthamoeba infection |
| Meningoencephalitis amoebic |
| Hepatic amoebiasis |
| Acanthamoeba keratitis |
| Balamuthia infection |
| Amoeboma |
| Babesiosis |
| Balantidiasis |
| Blastocystis infection |
| Cryptosporidiosis infection |
| Gastroenteritis cryptosporidial |
| Biliary tract infection cryptosporidial |
| Cyclosporidium infection |
| Giardiasis |
| Isosporiasis |
| Blackwater fever |
| Congenital malaria |
| Malaria |
| Plasmodium falciparum infection |
| Plasmodium malariae infection |
| Plasmodium ovale infection |
| Plasmodium vivax infection |
| Malarial myocarditis |
| Cerebral malaria |
| Plasmodium knowlesi infection |
| Malaria relapse |
| Malaria recrudescence |
| Algid malaria |
| Infection protozoal |
| Vorticella infection |
| Microsporidia infection |
| Protozoal corneal ulcer |
| Encephalitis protozoal |
| Gastrointestinal protozoal infection |
| Protothecosis |
| Sarcocystis infection |
| Cervicitis trichomonal |
| Prostatitis trichomonal |
| Trichomoniasis |
| Trichomoniasis intestinal |
| Urethritis trichomonal |
| Urogenital trichomoniasis |
| Vulvovaginitis trichomonal |
| Dientamoeba infection |
| African trypanosomiasis |
| American trypanosomiasis |
| Meningitis trypanosomal |
| Trypanosomiasis |
| Chagoma |
| Romana's sign |
| Chagas' cardiomyopathy |
| Congenital Chagas disease |
| Cutaneous leishmaniasis |
| Disseminated leishmaniasis |
| Leishmaniasis |
| Visceral leishmaniasis |
| Mucocutaneous leishmaniasis |
| Congenital toxoplasmosis |
| Eye infection toxoplasmal |
| Hepatitis toxoplasmal |
| Myocarditis toxoplasmal |
| Toxoplasmosis |
| Meningitis toxoplasmal |
| Cerebral toxoplasmosis |
| Pneumonia toxoplasmal |
| Disseminated toxoplasmosis |
| Endocarditis Q fever |
| Q fever |
| Coxiella infection |
| Human ehrlichiosis |
| Rickettsioses not tick borne |
| Encephalitis rickettsial |
| Rickettsiosis |
| Human anaplasmosis |
| Boutonneuse fever |
| North Asian tick typhus |
| Queensland tick typhus |
| Rickettsialpox |
| Rocky mountain spotted fever |
| Japanese spotted fever |
| Epidemic typhus |
| Murine typhus |
| Recrudescent typhus |
| Scrub typhus |
| Typhus |
| Adenoviral conjunctivitis |
| Adenoviral upper respiratory infection |
| Gastroenteritis adenovirus |
| Pharyngoconjunctival fever of children |
| Pneumonia adenoviral |
| Adenoviral hepatitis |
| Adenoviral haemorrhagic cystitis |
| Adenovirus infection |
| Adenovirus encephalomyeloradiculitis |
| Adenoviral encephalitis |
| Adenoviral meningitis |
| Adenovirus reactivation |
| Adenovirus interstitial nephritis |
| Encephalitis eastern equine |
| Encephalitis venezuelan equine |
| Encephalitis western equine |
| Epidemic polyarthritis |
| Chikungunya virus infection |
| Pogosta disease |
| Alphaviral infection |
| O'nyong-nyong fever |
| Arenaviral haemorrhagic fever |
| Argentine haemorrhagic fever |
| Bolivian haemorrhagic fever |
| Choriomeningitis lymphocytic |
| Lassa fever |
| Lujo haemorrhagic fever |
| Gastroenteritis astroviral |
| Gastroenteritis caliciviral |
| Gastroenteritis norovirus |
| Gastroenteritis sapovirus |
| Norovirus infection |
| Coxsackie carditis |
| Coxsackie endocarditis |
| Coxsackie myocarditis |
| Coxsackie pericarditis |
| Coxsackie viral disease of the newborn |
| Coxsackie viral infection |
| Epidemic pleurodynia |
| Herpangina |
| Meningitis coxsackie viral |
| Eczema Coxsackium |
| Coxsackie bronchitis |
| Congenital cytomegalovirus infection |
| Cytomegalovirus hepatitis |
| Cytomegalovirus infection |
| Cytomegalovirus mononucleosis |
| Encephalitis cytomegalovirus |
| Pneumonia cytomegaloviral |
| Cytomegalovirus chorioretinitis |
| Cytomegalovirus colitis |
| Cytomegalovirus duodenitis |
| Cytomegalovirus enterocolitis |
| Cytomegalovirus gastritis |
| Cytomegalovirus oesophagitis |
| Cytomegalovirus enteritis |
| Disseminated cytomegaloviral infection |
| Cytomegalovirus pancreatitis |
| Cytomegalovirus gastroenteritis |
| Cytomegalovirus urinary tract infection |
| Cytomegalovirus gastrointestinal infection |
| Cytomegalovirus myocarditis |
| Cytomegalovirus syndrome |
| Cytomegalovirus pericarditis |
| Cytomegalovirus infection reactivation |
| Cytomegalovirus viraemia |
| Cytomegalovirus mucocutaneous ulcer |
| Cytomegalovirus myelomeningoradiculitis |
| Cytomegalovirus gastrointestinal ulcer |
| Cytomegalovirus nephritis |
| Meningitis echo viral |
| Echo virus infection |
| Boston exanthema |
| Epstein-Barr virus infection |
| Epstein-Barr virus infection reactivation |
| Hepatitis infectious mononucleosis |
| Infectious mononucleosis |
| Oral hairy leukoplakia |
| Post transplant lymphoproliferative disorder |
| Epstein-Barr viraemia |
| X-linked lymphoproliferative syndrome |
| Epstein-Barr virus associated lymphoproliferative disorder |
| Epstein-Barr virus associated lymphoma |
| Epstein Barr virus positive mucocutaneous ulcer |
| Chronic active Epstein-Barr virus infection |
| Hantavirus pulmonary infection |
| Hantaviral infection |
| Haemorrhagic fever with renal syndrome |
| Congenital varicella infection |
| Eczema herpeticum |
| Exanthema subitum |
| Genital herpes |
| Herpes simplex |
| Congenital herpes simplex infection |
| Herpes simplex encephalitis |
| Herpes simplex meningitis |
| Herpes simplex otitis externa |
| Herpes simplex visceral |
| Herpes virus infection |
| Herpes zoster |
| Human herpesvirus 6 infection |
| Meningitis herpes |
| Meningoencephalitis herpetic |
| Ophthalmic herpes zoster |
| Pneumonia herpes viral |
| Proctitis herpes |
| Varicella |
| Herpes simplex virus conjunctivitis neonatal |
| Colitis herpes |
| Gastritis herpes |
| Herpes oesophagitis |
| Disseminated neonatal herpes simplex |
| Meningoencephalitis herpes simplex neonatal |
| Neonatal mucocutaneous herpes simplex |
| Herpes sepsis |
| Herpes zoster infection neurological |
| Herpes ophthalmic |
| Herpes dermatitis |
| Herpes zoster oticus |
| Varicella post vaccine |
| Human herpesvirus 7 infection |
| Herpes zoster disseminated |
| Herpes simplex pneumonia |
| Necrotising herpetic retinopathy |
| Human herpesvirus 8 infection |
| Human herpesvirus 6 infection reactivation |
| Herpes pharyngitis |
| Oral herpes |
| Herpes simplex hepatitis |
| Genital herpes zoster |
| Genital herpes simplex |
| Ophthalmic herpes simplex |
| Herpes simplex colitis |
| Herpes simplex gastritis |
| Varicella zoster gastritis |
| Herpes simplex oesophagitis |
| Varicella zoster oesophagitis |
| Herpes simplex pharyngitis |
| Herpes zoster pharyngitis |
| Herpes simplex sepsis |
| Herpes simplex meningoencephalitis |
| Herpes zoster meningoencephalitis |
| Meningomyelitis herpes |
| Herpes simplex meningomyelitis |
| Herpes zoster meningomyelitis |
| Herpes simplex necrotising retinopathy |
| Herpes zoster necrotising retinopathy |
| Varicella zoster pneumonia |
| Herpes zoster meningitis |
| Herpes zoster cutaneous disseminated |
| Varicella zoster sepsis |
| Nasal herpes |
| Varicella zoster virus infection |
| Disseminated varicella zoster vaccine virus infection |
| Lower respiratory tract herpes infection |
| Herpes simplex cervicitis |
| Varicella keratitis |
| Haemorrhagic varicella syndrome |
| Herpes zoster meningoradiculitis |
| Herpes simplex reactivation |
| Herpes simplex viraemia |
| Herpes zoster reactivation |
| Human herpesvirus 6 encephalitis |
| Herpetic radiculopathy |
| Kaposi sarcoma inflammatory cytokine syndrome |
| Disseminated varicella |
| Disseminated varicella zoster virus infection |
| Herpes simplex bronchitis |
| Oral herpes zoster |
| Varicella encephalitis |
| Varicella meningitis |
| Elsberg syndrome |
| Varicella zoster viraemia |
| Herpes simplex virus urethritis |
| Influenza |
| Pneumonia influenzal |
| Encephalitis influenzal |
| Avian influenza |
| H1N1 influenza |
| H2N2 influenza |
| H3N2 influenza |
| Molluscum contagiosum |
| Encephalitis mumps |
| Epididymitis mumps |
| Hepatitis mumps |
| Meningitis mumps |
| Mumps |
| Orchitis mumps |
| Pancreatitis mumps |
| Polyneuropathy mumps |
| Mumps deafness |
| Newcastle disease |
| Laryngeal papilloma |
| Penile wart |
| Respiratory papilloma |
| Skin papilloma |
| Urethral papilloma |
| Vulvovaginal warts |
| Cervicitis human papilloma virus |
| Epidermodysplasia verruciformis |
| Blepharal papilloma |
| Anogenital warts |
| Buschke-Lowenstein's tumour |
| Papilloma viral infection |
| Cervix warts |
| Vulvovaginal human papilloma virus infection |
| Congenital condyloma |
| Tracheal papilloma |
| Oral papilloma |
| Sinonasal papilloma |
| Anorectal human papilloma virus infection |
| Focal epithelial hyperplasia |
| Viral acanthoma |
| Parainfluenzae viral laryngotracheobronchitis |
| Pneumonia parainfluenzae viral |
| Parainfluenzae virus infection |
| Parainfluenzae viral bronchitis |
| Bovine pustular stomatitis virus infection |
| Orf |
| Milker's nodules |
| Parapox virus infection |
| Paravaccinia |
| Erythema infectiosum |
| Parvovirus B19 infection |
| Parvovirus infection |
| Parvovirus B19 infection reactivation |
| Human bocavirus infection |
| Congenital parvovirus B19 infection |
| Phlebotomus fever |
| Rift Valley fever |
| Severe fever with thrombocytopenia syndrome |
| Heartland virus infection |
| Bulbar poliomyelitis |
| Polioencephalitis |
| Poliomyelitis |
| Vaccine associated paralytic poliomyelitis |
| Creutzfeldt-Jakob disease |
| Kuru |
| Slow virus infection |
| Variant Creutzfeldt-Jakob disease |
| Gerstmann Straussler Scheinker syndrome |
| Fatal familial insomnia |
| Prion disease |
| Rabies |
| Pneumonia respiratory syncytial viral |
| Respiratory syncytial virus bronchiolitis |
| Respiratory syncytial virus infection |
| Respiratory syncytial virus bronchitis |
| Acquired immunodeficiency syndrome |
| Acute HIV infection |
| AIDS related complex |
| Asymptomatic HIV infection |
| Congenital HIV infection |
| Enterocolitis AIDS |
| HIV infection |
| HIV infection CDC Group I |
| HIV infection CDC Group II |
| HIV infection CDC Group III |
| HIV infection CDC Group IV subgroup A |
| HIV infection CDC Group IV subgroup B |
| HIV infection CDC Group IV subgroup C1 |
| HIV infection CDC Group IV subgroup C2 |
| HIV infection CDC Group IV subgroup D |
| HIV infection CDC Group IV subgroup E |
| HIV-2 infection |
| Human T-cell lymphotropic virus type I infection |
| Kaposi's sarcoma |
| Kaposi's sarcoma AIDS related |
| Lymphoma AIDS related |
| T-cell lymphoma |
| T-cell type acute leukaemia |
| Tropical spastic paresis |
| Persistent generalised lymphadenopathy |
| End stage AIDS |
| HIV wasting syndrome |
| AIDS retinopathy |
| Human T-cell lymphocytic virus type II infection |
| Retroviral infection |
| HIV infection CDC group IV |
| AIDS related complication |
| Human T-cell lymphotropic virus infection |
| HIV infection WHO clinical stage I |
| HIV infection WHO clinical stage II |
| HIV infection WHO clinical stage III |
| HIV infection WHO clinical stage IV |
| HIV peripheral neuropathy |
| Retroviral rebound syndrome |
| AIDS cholangiopathy |
| HIV cardiomyopathy |
| HIV enteropathy |
| HIV associated nephropathy |
| Perinatal HIV infection |
| Immune reconstitution inflammatory syndrome associated Kaposi's sarcoma |
| HIV infection CDC category A |
| HIV infection CDC category B |
| HIV infection CDC category C |
| HIV viraemia |
| HIV-associated neurocognitive disorder |
| HIV lipodystrophy |
| CSF HIV escape syndrome |
| Papular pruritic eruption of HIV |
| HIV meningoencephalitis |
| AIDS dysmorphic syndrome |
| Rhinovirus infection |
| Gastroenteritis rotavirus |
| Rotavirus infection |
| Arthritis rubella |
| Cataract congenital |
| Congenital rubella infection |
| Encephalomyelitis rubella |
| Rubella |
| Rubella in pregnancy |
| Rubella infection neurological |
| Congenital rubella syndrome |
| Keratoconjunctivitis measles |
| Measles |
| Otitis media post measles |
| Pneumonia measles |
| Measles post vaccine |
| Post measles blindness |
| Measles meningitis |
| Arthritis viral |
| Bronchiolitis |
| Conjunctivitis viral |
| Encephalitis viral |
| Eye infection viral |
| Foot and mouth disease |
| Gastroenteritis viral |
| Hepatitis viral |
| Laryngitis viral |
| Meningitis viral |
| Pneumonia viral |
| Retinitis viral |
| Sweating fever |
| Vestibular neuronitis |
| Viral infection |
| Viral labyrinthitis |
| Viral myocarditis |
| Viral pericarditis |
| Viral pharyngitis |
| Viral rash |
| Viral tonsillitis |
| Viral tracheitis |
| Viral upper respiratory tract infection |
| Viral diarrhoea |
| Viral myositis |
| Viral sinusitis |
| Gastritis viral |
| Pleurisy viral |
| Bronchitis viral |
| Peritonitis viral |
| Gianotti-Crosti syndrome |
| Viral corneal ulcer |
| Viral vasculitis |
| Post viral fatigue syndrome |
| Vaginitis viral |
| Viral haemorrhagic cystitis |
| Viral oesophagitis |
| Central nervous system viral infection |
| Haemorrhagic fever |
| Tracheobronchitis viral |
| Ear infection viral |
| Endocarditis viral |
| Enterocolitis viral |
| Keratitis viral |
| Respiratory tract infection viral |
| Asymptomatic viral hepatitis |
| Nipah virus infection |
| Urinary tract infection viral |
| Viral rhinitis |
| Viral skin infection |
| Otitis media viral |
| Otitis externa viral |
| Lower respiratory tract infection viral |
| Pancreatitis viral |
| Splenic infection viral |
| Cystitis viral |
| Biliary tract infection viral |
| Lymphadenitis viral |
| Pyelonephritis viral |
| Genital infection viral |
| Oral viral infection |
| Osteomyelitis viral |
| Wound infection viral |
| Nail bed infection viral |
| Metapneumovirus infection |
| Superinfection viral |
| Eruptive pseudoangiomatosis |
| Viral cardiomyopathy |
| Gastrointestinal viral infection |
| Viral uveitis |
| Withdrawal hepatitis |
| Viral sepsis |
| Parechovirus infection |
| Viral myelitis |
| Viral parotitis |
| Viral mastitis |
| Meningoencephalitis viral |
| Viral epiglottitis |
| Picornavirus infection |
| Systemic viral infection |
| Viral keratouveitis |
| Congenital viral hepatitis |
| Metapneumovirus bronchiolitis |
| Metapneumovirus pneumonia |
| Encephalomyelitis viral |
| Viruria |
| Borna virus infection |
| Viral abdominal infection |
| Congenital viral infection |
| Ebola disease |
| Marburg disease |
| Ebola Reston virus infection |
| Filovirus infection |
| Congenital Ebola virus infection |
| Colorado tick fever |
| Congo-Crimean haemorrhagic fever |
| Encephalitis california |
| Orbivirus infection |
| Arboviral infection |
| Snowshoe hare virus infection |
| Oropouche fever |
| Reoviral infection |
| Jamestown Canyon virus infection |
| Jamestown Canyon encephalitis |
| Dengue fever |
| Encephalitis australia |
| Encephalitis Japanese B |
| Kyasanur Forest disease |
| Louping ill |
| Omsk haemorrhagic fever |
| St. Louis encephalitis |
| Tick-borne viral encephalitis |
| Yellow fever |
| Murray Valley encephalitis |
| Flavivirus infection |
| Dengue haemorrhagic fever |
| West Nile viral infection |
| Yellow fever vaccine-associated viscerotropic disease |
| Yellow fever vaccine-associated neurotropic disease |
| Zika virus infection |
| Rocio virus infection |
| Congenital Zika syndrome |
| Zika virus associated ocular birth defect |
| Zika virus associated Guillain Barre syndrome |
| Zika virus associated birth defect |
| Zika virus associated microencephaly |
| Alongshan virus infection |
| Congenital dengue disease |
| Breakthrough dengue fever |
| Dengue shock syndrome |
| JC virus infection |
| Progressive multifocal leukoencephalopathy |
| BK virus infection |
| Human polyomavirus infection |
| Polyomavirus-associated nephropathy |
| JC virus granule cell neuronopathy |
| Polyomavirus viraemia |
| Merkel cell polyomavirus infection |
| WU virus infection |
| Cow pox |
| Smallpox |
| Vaccinia virus infection |
| Eczema vaccinatum |
| Post vaccination autoinoculation |
| Monkeypox |
| Generalised vaccinia |
| Progressive vaccinia |
| Orthopox virus infection |
| Enterovirus infection |
| Gastroenteritis enteroviral |
| Hand-foot-and-mouth disease |
| Meningitis enteroviral |
| Central nervous system enteroviral infection |
| Encephalitis enteroviral |
| Acute haemorrhagic conjunctivitis |
| Enterovirus myocarditis |
| Coronavirus infection |
| Severe acute respiratory syndrome |
| Middle East respiratory syndrome |
| COVID-19 |
| COVID-19 pneumonia |
| Coronavirus pneumonia |
| Suspected COVID-19 |
| Asymptomatic COVID-19 |
| SARS-CoV-2 sepsis |
| SARS-CoV-2 viraemia |
| HCoV-229E infection |
| HCoV-OC43 infection |
| HCoV-NL63 infection |
| HCoV-HKU1 infection |
| Congenital COVID-19 |
| Vaccine derived SARS-CoV-2 infection |
| Post-acute COVID-19 syndrome |
| Breakthrough COVID-19 |
| Chronic hepatitis B |
| Chronic hepatitis C |
| Congenital hepatitis B infection |
| Hepatitis A |
| Hepatitis B |
| Hepatitis C |
| Hepatitis D |
| Hepatitis E |
| Hepatitis F |
| Hepatitis G |
| Hepatitis H |
| Hepatitis non-A non-B |
| Hepatitis non-A non-B non-C |
| Hepatitis B reactivation |
| Acute hepatitis B |
| Acute hepatitis C |
| Hepatitis virus-associated nephropathy |
| Perinatal HBV infection |
| Congenital hepatitis C infection |

| Supplementary Table 2 : CYP inhibitors and their action on main CYP450 implicated in clozapine metabolism | | | | | | | | | | |
| --- | --- | --- | --- | --- | --- | --- | --- | --- | --- | --- |
| CYP inhibitor | CYP1A2 | | CYP3A4 | | CYP2D6 | | CYP2C9 | | CYP2C19 | |
|  | WHO | HUG | WHO | HUG | WHO | HUG | WHO | HUG | WHO | HUG |
| Amiodarone | UN | MO | WE | MO | WE | ST | MO | ST | - | - |
| Atazanavir | UN | MO | - | ST | - | - | UN | MO | - | - |
| Cannabidiol | - | MO | - | ST | - | - | - | - | - | - |
| Ciclosporin | - | - | MO | ST | - | - | - | - | - | - |
| Clarithromycin | - | - | - | ST | - | - | - | - | - | - |
| Clobazam | - | - | - | MO | WE | - | - | - | - | MO |
| Esomeprazole | - | - | - | - | - | - | - | - | MO | ST |
| Felbamate | - | - | - | - | - | - | - | - | WE | ST |
| Fluconazole | - | - | MO | ST | - | - | MO | ST | ST | ST |
| Fluvoxamine | ST | ST | MO | MO | UN | - | WE | MO | ST | ST |
| Itraconazole | - | - | ST | ST | - | - | - | - | - | - |
| Lansoprazole | - | - | - | - | - | - | - | - | UN | ST |
| Metronidazole | - | - | - | - | - | - | WE | ST | - | - |
| Omeprazole | - | - | - | - | - | - | - | - | MO | ST |
| Ritonavir | UN | - | ST | ST | WE | ST | UN | - | - | - |
| Valproic acid | - | - | - | - | - | - | UN | ST | - | - |
| Voriconazole | - | - | ST | ST | - | - | WE | ST | MO | ST |
| CYP: cytochrome. FDA: Food and Drug Administration, HUG: Hôpital Universitaire de Genève (Geneva Universitary Hospital), MO: moderate inhibitor, ST: strong inhibitor, UN: unclassified inhibitor, WE: weak inhibitor, WHO: World Health Organization classification.  HUG classification contains 2 levels: MO, ST  WHO classification contains 4 levels: UN, WE, MO, ST | | | | | | | | | | |

**Supplementary Table 4**. Disproportionality analysis by dose quartile of clozapine-associated infections

| Quartile | N | IC | IC025 | IC975 |
| --- | --- | --- | --- | --- |
| Q1 | 663 | 1.18 | 1.05 | 1.27 |
| Q2 | 542 | 0.75 | 0.60 | 0.85 |
| Q3 | 646 | 0.88 | 0.75 | 0.97 |
| Q4 | 468 | 1.21 | 1.06 | 1.32 |
| N: Number of reports, IC, Information Component, IC025: lower end of the credibility interval. IC975: upper end of the credibility interval | | | | |

**Supplementary Table 5**. Terms of infection associated with a significant over-reporting with clozapine (less than 15 reports per term)

| Term | | | N | | IC | IC025 | IC975 |
| --- | --- | --- | --- | --- | --- | --- | --- |
| Complicated appendicitis | | | 9 | | 3.18 | 2.04 | 3.94 |
| Viral pericarditis | | | 7 | | 2.49 | 1.19 | 3.34 |
| Biliary sepsis | | | 8 | | 2.08 | 0.87 | 2.89 |
| Subacute endocarditis | | | 5 | | 2.40 | 0.84 | 3.39 |
| Appendiceal abscess | | | 6 | | 2.22 | 0.81 | 3.14 |
| Infection parasitic | | | 11 | | 1.45 | 0.42 | 2.14 |
| Lower respiratory tract infection viral | | | 8 | | 1.56 | 0.35 | 2.36 |
| Pneumonia necrotising | | | 7 | | 1.62 | 0.32 | 2.47 |
| Febrile infection | | | 14 | | 1.2 | 0.3 | 1.83 |
| Tracheobronchitis | | | 10 | | 1.37 | 0.3 | 2.1 |
| Diarrhoea infectious | | | 9 | | 1.4 | 0.26 | 2.16 |
| Breast abscess | | | 13 | | 1.16 | 0.22 | 1.8 |
| Pilonidal disease | | | 10 | | 1.29 | 0.21 | 2.01 |
| Orchitis | | | 13 | | 1.07 | 0.13 | 1.71 |
| 0.37 | 0.09 | 0.57 | |  |  |  |  |
| Groin abscess | | | 13 | | 1.02 | 0.08 | 1.66 |
| Pneumonia viral | | | 23 | | 0.77 | 0.07 | 1.26 |
| Pneumonia streptococcal | | | 11 | | 1.09 | 0.07 | 1.79 |
| Bronchitis viral | | | 9 | | 1.2 | 0.06 | 1.96 |
| Mononucleosis syndrome | | | 5 | | 1.62 | 0.05 | 2.6 |
| Pyuria | | | 13 | | 0.94 | 0 | 1.58 |
| N : Number of reports. IC ; Information Component. IC025 : lower end of the credibility interval. IC975: upper end of the credibility interval | | | | | | | |
